# Sea-ATI unravels novel vocabularies of plant active cistrome

**DOI:** 10.1093/nar/gkad853

**Published:** 2023-10-18

**Authors:** Chenjin Wen, Zhen Yuan, Xiaotian Zhang, Hao Chen, Lin Luo, Wanying Li, Tian Li, Nana Ma, Fei Mao, Dongmei Lin, Zhanxi Lin, Chentao Lin, Tongda Xu, Peitao Lü, Juncheng Lin, Fangjie Zhu

**Affiliations:** College of Life Science, Haixia Institute of Science and Technology, National Engineering Research Center of JUNCAO, Fujian Agriculture and Forestry University, Fuzhou 350002, Fujian, China; College of Life Science, Haixia Institute of Science and Technology, National Engineering Research Center of JUNCAO, Fujian Agriculture and Forestry University, Fuzhou 350002, Fujian, China; College of Life Science, Haixia Institute of Science and Technology, National Engineering Research Center of JUNCAO, Fujian Agriculture and Forestry University, Fuzhou 350002, Fujian, China; College of Life Science, Haixia Institute of Science and Technology, National Engineering Research Center of JUNCAO, Fujian Agriculture and Forestry University, Fuzhou 350002, Fujian, China; College of Life Science, Haixia Institute of Science and Technology, National Engineering Research Center of JUNCAO, Fujian Agriculture and Forestry University, Fuzhou 350002, Fujian, China; College of Life Science, Haixia Institute of Science and Technology, National Engineering Research Center of JUNCAO, Fujian Agriculture and Forestry University, Fuzhou 350002, Fujian, China; College of Life Science, Haixia Institute of Science and Technology, National Engineering Research Center of JUNCAO, Fujian Agriculture and Forestry University, Fuzhou 350002, Fujian, China; College of Life Science, Haixia Institute of Science and Technology, National Engineering Research Center of JUNCAO, Fujian Agriculture and Forestry University, Fuzhou 350002, Fujian, China; College of Life Science, Haixia Institute of Science and Technology, National Engineering Research Center of JUNCAO, Fujian Agriculture and Forestry University, Fuzhou 350002, Fujian, China; College of Life Science, Haixia Institute of Science and Technology, National Engineering Research Center of JUNCAO, Fujian Agriculture and Forestry University, Fuzhou 350002, Fujian, China; College of Life Science, Haixia Institute of Science and Technology, National Engineering Research Center of JUNCAO, Fujian Agriculture and Forestry University, Fuzhou 350002, Fujian, China; College of Life Science, Haixia Institute of Science and Technology, National Engineering Research Center of JUNCAO, Fujian Agriculture and Forestry University, Fuzhou 350002, Fujian, China; College of Life Science, Haixia Institute of Science and Technology, National Engineering Research Center of JUNCAO, Fujian Agriculture and Forestry University, Fuzhou 350002, Fujian, China; College of Horticulture, Fujian Agriculture and Forestry University, Fuzhou 350002, Fujian, China; College of Life Science, Haixia Institute of Science and Technology, National Engineering Research Center of JUNCAO, Fujian Agriculture and Forestry University, Fuzhou 350002, Fujian, China; College of Life Science, Haixia Institute of Science and Technology, National Engineering Research Center of JUNCAO, Fujian Agriculture and Forestry University, Fuzhou 350002, Fujian, China

## Abstract

The cistrome consists of all *cis-*acting regulatory elements recognized by transcription factors (TFs). However, only a portion of the cistrome is active for TF binding in a specific tissue. Resolving the active cistrome in plants remains challenging. In this study, we report the assay sequential extraction assisted-active TF identification (sea-ATI), a low-input method that profiles the DNA sequences recognized by TFs in a target tissue. We applied sea-ATI to seven plant tissues to survey their active cistrome and generated 41 motif models, including 15 new models that represent previously unidentified *cis-*regulatory vocabularies. ATAC-seq and RNA-seq analyses confirmed the functionality of the *cis-*elements from the new models, in that they are actively bound *in vivo*, located near the transcription start site, and influence chromatin accessibility and transcription. Furthermore, comparing dimeric WRKY CREs between sea-ATI and DAP-seq libraries revealed that thermodynamics and genetic drifts cooperatively shaped their evolution. Notably, sea-ATI can identify not only positive but also negative regulatory *cis-*elements, thereby providing unique insights into the functional non-coding genome of plants.

## Graphical Abstract

**Graphical Abstract:**
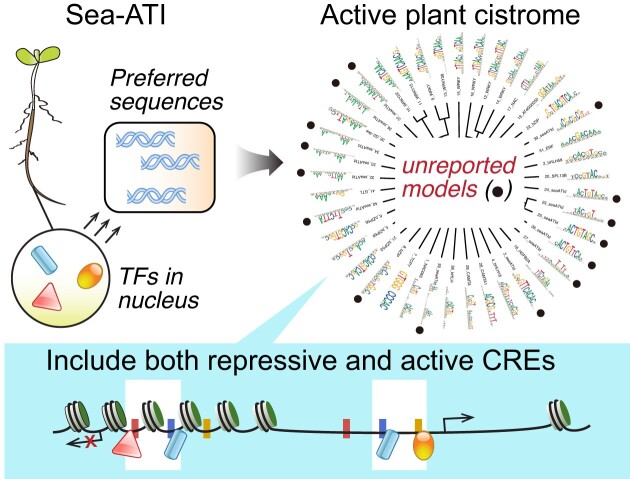

## Introduction

The fast-expanding repertoire of plant genomes has opened up numerous opportunities for researchers to investigate gene function and evolution (1,2). However, understanding how genes are expressed in intricate spatiotemporal patterns remains a major challenge in the post-genomic era. To tackle this challenge, researchers must decipher the ‘dark matter’—the noncoding genome (3)—by identifying *cis*-regulatory elements (CREs) that serve as binding sites for regulatory proteins and constructing a complete cistrome (4). TF-centered approaches have been successful in resolving CREs. ChIP-seq (5,6), and recently CUT&Tag (7), use antibodies to detect natural binding sites of TFs. While these methods yield high-fidelity CREs, they are technically demanding and heavily reliant on antibody quality. To overcome these limitations, *in vitro* methods such as PBM (8,9), SELEX (10) and DAP-seq (11–13), along with *semi-in vivo* ChIP-seq using epitope-tagged TFs (14), have been widely adopted for rapid, low-cost, and scalable profiling of CREs recognized by a TF. These assays can be massively parallelized to generate CRE libraries for hundreds of TFs. Although the TF-centered approaches have produced rich datasets regarding CREs and CRE models of TFs (15–17), and collectively delineated regions of the complete cistrome, examining one TF at a time is insufficient to obtain information on the active cistrome—the binding sites of all active TFs that are responsible for the localized transcriptional program in a specific tissue.

Alternatively, genome-wide annotations of the active cistrome can be achieved through open-chromatin profiling techniques such as ATAC-seq (18), DNase-seq (19), and MH-seq (20). These methods provide a comprehensive landscape of the active cistrome within a tissue of interest. However, the annotated open chromatin regions have limited resolution, ranging from hundreds to thousands of base pairs in width. Further narrowing down to the sites of the enriched or known motifs in the open chromatin yields CREs with a higher resolution but poses a risk of false positives. This is because the binding sites of TFs tend to cluster. When only one TF is active, it can create open chromatin regions harboring CREs of many inactive TFs. Moreover, these approaches cannot identify repressive CREs that decrease chromatin accessibility upon binding.

Two powerful approaches to profile the active cistrome are STARR-seq (self-transcribing active regulatory region sequencing) (21) and ATI (active TF identification) (22). STARR-seq evaluates millions of candidate sequences for their enhancer activities, depicting the transcriptionally active cistrome. In contrast, the ATI assay enriches for sequences directly bound by TFs in cellular nuclear extracts, illustrating the biochemically active cistrome. The biochemically active CREs may not necessarily be the most effective transcriptional activators but are strong predictors of chromatin accessibility (22,23). Thus, STARR-seq and ATI complement each other to give a comprehensive view of the active cistrome. For plant samples, STARR-seq has been established (24–27), but applications of ATI are yet to be explored.

Because the nuclei isolation step in ATI is inefficient for plant samples, here we establish sequential extraction assisted-active TF identification (sea-ATI), a low-input method suitable for profiling the active cistrome in plants. Sea-ATI bypasses the inefficient nuclei isolation by sequential extractions that separate cytosolic components and nuclear contents. Surprisingly, applying sea-ATI to plant tissues identified large amounts of previously unreported binding specificities of CREs. CREs with the novel specificities are then validated as functional due to their involvement in biochemical binding, epigenetic regulation, and transcriptional control. The results demonstrated the applications of sea-ATI and indicate that physiological *cis-*regulatory syntax in plants is yet far from completely deciphered.

## Materials and methods

### Plant growth and sample collection

Cotyledons and roots: *A. thaliana* (Col-0) seeds were surface sterilized and synchronized at 4°C for 3 days, and then sown on 1/2 MS (2.22 g/l MS medium, 10 g/l sucrose, 0.5 g/l MES, 8 g/l agar, pH 5.9) at 22°C under 16 h light/8 h dark photoperiod. Cotyledons and roots were separated from 5-day-old seedlings.

Stems and siliques: *A. thaliana* (Col-0) was planted in charcoal soil mixed with vermiculite in a 3:1 ratio. At 22°C under 16 h light/8 h dark photoperiod. The tender stems were collected from 5-week-old plants. Siliques were collected from 8-week-old plants.

PSB-D cells: *A. thaliana* (Ler) suspension cell line PSB-D are cultured in MS medium in 250 ml flask at 25°C in a light-protected shaker at 130 rpm, and subcultured once per 5 days by transferring 2.5 ml suspension of old cells into 50 ml of fresh medium. To harvest PSB-D cells, 50 ml suspension was centrifuged at 800 g for 3 min. The precipitate is collected and washed once with 1 × PBS.

Calluses: *A. thaliana* (Col-0) seeds were surface sterilized and synchronized at 4°C for 3 days. Treated seeds were sown on 1/2 MS plates. After one day of growth with light (16 h light/8 h dark, 22°C), plates were kept in the dark for ∼5 days to promote the growth of the hypocotyl. The hypocotyls were cut into 2∼3 cm long segments by scissors and inoculated onto the callus induction media (3.21 g/l B5 medium, 20 g/l glucose, 0.5 g/l MES, 8 g/l agar, 2.2 μM 2,4-D and 0.2 μM KT) for 14 days. The outgrown calluses were then collected.

*C.f*. shoots: *Cenchrus fungigraminus* were propagated with nodes and cultivated in seedling trays for 20 days, then transplanted into fields at the germplasm resource nursery of the National Engineering Research Center of JUNCAO Technology. All the plants were watered with natural rainfall and fertilized with 2 g of compound fertilizer (FuJian AoLiGaoTa Fertilizer Co., Ltd, Zhangzhou, China). The tillers emerging from the base with a length of 10 ± 5 cm and not affected by diseases and pests were collected in the summer of 2022.

### Sequential extraction assisted-active TF identification (sea-ATI)

For each sample, 0.05–0.1 g tissue was collected into a 2 ml centrifuge tube, flash frozen with liquid nitrogen, and ground into powder with 4 mm stainless steel beads at 50 Hz for 180 s by using a high-throughput tissue grinder (Wonbio-48R, Shanghai Jingxin). The PSB-D cells were ground with a pestle in liquid nitrogen. The ground samples were then placed on ice and washed twice with 1 ml chilled wash buffer (5 mM Tris–HCl, 10 mM EDTA, 1 mM DTT, pH 7.5) to remove cytosols. The precipitate was collected by centrifuging at 800 g for 1 min, and then resuspended with 300 μl nuclear extraction buffer (10 mM HEPES, 500 mM KCl, 1.5 mM MgCl_2_, 5 mM EDTA, 0.2% Triton X-100, 10% glycerol, pH 7.5) supplemented with protease inhibitors (1 tablet per 50 ml, A32965, Thermo Scientific), 1 mM DTT, and 100 μg/ml RNase A. The mixture was incubated at room temperature for 30 min on a rotating mixer, followed by centrifugation at 15 000 g for 10 min. The supernatant containing the extracted nuclear proteins was then collected for the subsequent ATI assay. To test the cytosolic proteins as a control, 300 μl chilled wash buffer was added into the ground tissue and after 2 min centrifuged to collect the supernatant.

The DNA ligands used in sea-ATI contain a 30-bp randomized region flanked by adaptors designed according to the Truseq and Nextera Illumina library (Supplementary Table 1). The length of the ligand is 76 bp in total. Single-stranded oligos of the sea-ATI ligands were synthesized by Sangong Biotech (Shanghai), and then double-stranded by PCR amplification with primers designed to pair with the fixed Illumina adaptors. The PCR product serves as a complex input library to select DNA ligands recognized by TFs in the nuclear extract. Next, 10 μl of the PCR product, 5 μl nuclear extract (∼5 μg), and 15 μl binding buffer (1 mM K_2_HPO_4_, 2 mM MgSO_4_, 100 μM EGTA, and 3 μM ZnSO_4_, in 20 mM HEPES, pH 7.5) were mixed and incubated at 25°C for 30 min. To separate TF-bound ligands from the unbound, the mixture was loaded to an 8% PAGE gel (pre-run at 150 V for 15 min) to perform the electrophoretic mobility shift assay (EMSA). The electrophoresis was run on ice with 106 V constant voltage for 1 h in 0.5× TBE buffer (1 mM EDTA, 45 mM Tris-borate, pH 8.0). After staining with SYBR green I fluorescent dye, the fragments above the free-DNA band were collected by slicing the gel, and eluted with 300 μl elution buffer (10 mM Tris–HCl, 0.2 mM EDTA, pH 8.0) at 65°C for 2 h with mixing to extract the DNA ligands. The elution containing bound ligands was then PCR amplified. This enrichment process was repeated for 3–5 cycles. The enriched libraries were further amplified with the PE primers (Supplementary Table 1) and sequenced.

### Pre-processing and motif discovery of sea-ATI

Raw data from Illumina sequencing were demultiplexed according to the i7 indices of each sample. The pre-processing filters out low-quality reads, trims the adaptors, and merges paired-end reads together. We use the tool fastp (28) for pre-processing, with the following parameters: -q 10 -m -a –overlap_len_require = 5 -gx –length_required = 15 –n_base_limit = 10 -y –omplexity_threshold = 5. PCR duplicates were then removed with an R script. The autoseed (29) program was used to *de novo* discover motifs from the pre-processed ATI reads, with a multinomial of 1, seed lengths ranging from 8 to 10, and 40 as the local-max count cut-off. The algorithm first identifies the enriched local-max kmers as seeds, and subsequently from the seeds derives the position frequency matrices (PFM) with a multinomial model (30). This method can separate closely related binding modes if the Hamming distance between the seeds is larger than or equal to two.

To curate a motif set from all examined plant tissues, we selected motifs with information content (IC) <1.8 for their consensus, total IC between 2 and 30, and per-base IC larger than 0.2. IC of the consensus or at each position of the motif matrix is calculated as


\begin{equation*}IC = \mathop \sum \limits_{N = A}^T \left( {p\left( N \right) \times lo{g}_2\left( {\frac{{p\left( N \right)}}{{0.25}}} \right)} \right)\end{equation*}

where *p(N)* denotes the probability of each nucleotide, and 0.25 is the background frequency in a random distribution. Total IC is the sum of IC over all positions of a motif. Per-base IC was calculated by dividing the total IC with the length of the motif. We used IC of the consensus as one of the criteria to filter out homonucleotide runs, which are frequently generated as non-specific biases in the assay. Next, motifs with Pearson's correlation coefficient larger than 0.90 were considered identical, and duplicates were removed with the one of the highest IC retained. The enrichment of all motifs in each sample was analyzed using motifmatchr, an R wrapper of MOODS (31).

Reported motifs of *A. thaliana* TFs from multiple sources were previously curated in PlantPAN (15) and PlantTFDB (16) databases. HOMER plants (32) reanalyzed the largest DAP-seq dataset (11) to create a library of nearly 500 motifs. To identify motif models not reported previously, each curated sea-ATI motif is compared to *A. thaliana* motifs from the three abovementioned datasets. Each sea-ATI motif was aligned against the reported motifs that are most similar (Supplementary Figure 1). The alignments were examined manually to classify sea-ATI motifs into three categories: distinct, similar, and reported (Supplementary Table 2). Distinct models are motifs that align poorly with reported ones, representing the newly identified specificities of the active cistrome. Reported models find good alignments with previous motifs. Similar models also align with the reported ones but with clear discrepancies. Distinct models are named ‘seaATId’ motifs. Reported and similar motif models are named after the best-aligned reported motifs. Only the family name is assigned when discrepancies exist. Classification and naming of TF families were in accord with PlantTFDB.

### Mutual information-based analyses of TF signals

To detect the enrichment of TF binding signals (i.e. the CREs) in sea-ATI libraries, we calculated mutual information (MI) measures between pairs of sub-sequences (33). MI analyses are suitable for sea-ATI because each library contains binding signals of multiple TFs. Whereas approaches relying on binding models of individual TFs only assess the enrichment of one TF at a time, MI analyses collectively capture binding events of all TFs to assess the overall signal strength. Moreover, MI analyses are convenient also because they operate independently of prior knowledge on TF binding. The rationale for MI measures to detect TF binding is that when a TF binds DNA, it will contact two non-overlapping 3-bp wide positions on the sea-ATI ligand. This increases the co-occurrence of TF-preferred sequences at the two 3-bp wide positions in the enriched sea-ATI library. The biased joint distribution between the 3-bp wide positions can then be captured by calculating the MI of 3-mer distributions at the positions. Because the footprints of most TFs are small (PFM width 7–21 bp), their binding will contact neighboring or closely spaced 3-bp wide positions. Thus, a library enriched with TF signals will show strong MI signals near the hypotenuse of the triangular plot, where MIs between continuous or closely spaced 3-bp positions are depicted.

First, to detect ubiquitous binding events of TFs in sea-ATI libraries, E-MI (enriched-sequence-based mutual information) was calculated as previously described (33). E-MI between 3-mer distributions at two non-overlapping positions is calculated by taking the sum of MIs over the top 10 most enriched 3-mer pairs:


\begin{eqnarray*}E - MI\left( {pos1,pos2} \right){\mathrm{\ }} = {\mathrm{\ }}\mathop \sum \limits_{\begin{array}{@{}*{1}{c}@{}} {top\ 10\ enriched\ } \\{3 + 3{\mathrm{ - }}mers} \end{array}}\\ P\left( {3{\mathrm{\ }} + {\mathrm{\ }}3{\mathrm{ - }}mer} \right)lo{g}_2\frac{{P\left( {3{\mathrm{\ }} + {\mathrm{\ }}3{\mathrm{ - }}mer} \right)}}{{{P}_{pos1}\left( {3{\mathrm{ - }}mer} \right){P}_{pos2}\left( {3{\mathrm{ - }}mer} \right)}}\end{eqnarray*}

where *P(3 + 3-mer)* is the actual probability of a 3-mer pair observed at position 1 and position 2, namely, the frequency of a gapped or continuous 6 mer. *P_pos1_(3-mer)* and *P_pos2_(3-mer)*, respectively, are the marginal probabilities of the constitutive 3-mers at position 1 and position 2. Their product in the denominator represents the expected probability of the 3-mer pair.

To adapt the MI analyses for the detection of dimeric binding signals, we employed the D-MI (dimeric-sequence-based mutual information) measures that sum up MIs from 3-mer pairs that are either identical or reverse-complement to each other, because biases will be specifically introduced into joint distributions between these 3-mer pairs when dimeric CRE sequences enrich in the library. D-MI is calculated as follows:


\begin{equation*}D - MI\left( {pos1,pos2} \right){\mathrm{\ }} = {\mathrm{\ }}\mathop \sum \limits_{\begin{array}{@{}*{1}{c}@{}} {dimeric\ }\\ {3\ + \ 3{\mathrm{ - }}mers} \end{array}}\\ P\left( {3{\mathrm{\ }} + {\mathrm{\ }}3{\mathrm{ - }}mer} \right)lo{g}_2\frac{{P\left( {3{\mathrm{\ }} + {\mathrm{\ }}3{\mathrm{ - }}mer} \right)}}{{{P}_{pos1}\left( {3{\mathrm{ - }}mer} \right){P}_{pos2}\left( {3{\mathrm{ - }}mer} \right)}}\end{equation*}

### Analyses of dimeric binding preferences

Motif models of WRKY dimers were discovered together with other models as mentioned above. The dimeric preference of WRKY regarding the relative monomer orientation and spacing was analyzed by counting occurrences of TTGAC (the core DNA sequence recognized by WRKY) concatenated with different relative orientations and spacings, and either in sea-ATI or DAP-seq libraries. To check whether two WRKY DBDs make protein-level contacts upon dimeric binding, structural models were constructed. First, B-DNA models harboring appropriately spaced and oriented TTGAC sequences were constructed using a web-based tool (http://www.scfbio-iitd.res.in/software/drugdesign/bdna.jsp), serving as the DNA scaffolds that contain a dimeric WRKY CRE. Next, the structure model of the WRKY-DNA complex was aligned with the B-DNA models. To make the alignments, the TTGAC ribonucleotides on the WRKY-DNA complex were superimposed with those on the B-DNA scaffolds by matching C1–C4 on all deoxyribose rings of the 5-bp DNA sequences. Then the DNA chains in the model of the WRKY-DNA complex were hidden. Alignment and visualization of the protein structures were performed with UCSF Chimera (34). The WRKY-DNA structure of PDB ID 6J4F was used in all alignments.

### Chromatin accessibility and footprints

For ATAC-seq library preparation, approximately 0.05 g of plant materials were collected. Tissues were homogenized by blade-chopping in ∼20 μl pre-chilled lysis buffer (15 mM Tris–HCl pH 7.5, 20 mM NaCl, 0.5 mM spermidine, 2 mM DTT and 0.2% Triton X-100) on ice. The slurry was collected into 1 ml lysis buffer and incubated at 4°C with gentle rotation for 30 min. The lysates were filtered by two layers of Miracloth and carefully loaded on 2 ml dense sucrose buffer (20 mM Tris–HCl pH 8.0, 2 mM MgCl_2_, 2 mM EDTA, 15 mM 2-ME, 1.7 M sucrose, 0.2% Triton X-100) in a 15 ml falcon tube. The assembled mixture was centrifuged at 2200 g and 4°C for 20 min. The pellet was resuspended by 1 ml nuclei washing buffer (10 mM Tris–HCl pH 8.0, 5 mM MgCl_2_) and centrifuged at 1200 g and 4°C for 5 min. The isolated nuclei were immediately used for tagging by Tn5 transposase (TD501, Vazyme Biotech) at 37°C for 30 min. The product was purified with two volumes of DNA purification beads (N411, Vazyme Biotech) and amplified by TruePrep Index Kit V2 for Illumina kit (TD202, Vazyme Biotech). PCR products longer than 180 bp were again purified by DNA purification beads and subjected to paired-end sequencing (2 × 150 bp).

For ATAC-seq data analyses, adaptor sequences were first removed using Trim Galore with parameters ‘-q 30 –paired –stringency 5 –fastqc –gzip’. The BWA aligner (35) was used to align the reads to the genome with the quality threshold set to 20. Next, samtools (36) was used to convert the alignments to bam format, and then used to sort and remove duplicates, and to index the genomic alignment files. MACS3 (37) was used to call peaks. TOBIAS (38) with default parameters was used to calculate the cutting profiles and footprint scores for the sea-ATI motifs. In TOBIAS workflow, the sequence bias of Tn5 cutting is first corrected; then the footprint depth for a set of aggregated CREs is defined as the difference of mean Tn5 cutting frequencies between the flanking region and the footprint region. Here the obtained footprint depth is further subtracted with the TOBIAS footprint of the CREs in a control library, where protein-free genomic DNA was tagged with Tn5. This is because CRE footprints could still be visible, though much weaker, in the control library after the TOBIAS bias correction (Supplementary Figure 3C). Visualizations were generated with custom R scripts using the ComplexHeatmap package (39).

For MNase-seq library preparation, into 0.05 g homogenized plant tissue, 1 ml chilled RIPA buffer (10 mM Tris–HCl pH 8.0, 140 mM NaCl, 1 mM EDTA, 1% Triton X-100, 0.1% SDS, 0.1% sodium deoxycholate, 0.25 M sucrose, 0.1% beta-ME, 1 mM PMSF) was added. After a thorough vortexing, the mixture is centrifuged at 800 g for 2 min to remove the supernatant. The washing process is repeated 3–5 times. The final pellet containing nuclei was resuspended by 200–800 μl MNase buffer (0.3 M sucrose, 20 mM Tris–HCl pH 7.5, 3 mM CaCl_2_). The Micrococcal Nuclease (M0247S, 300 U/μl, New England Biolabs) was diluted 128 times with MNase buffer, and 6 μl of the diluted MNase was added into 300 μl resuspension of the nuclei, incubated at 37°C and 1500 rpm for 8 min on a mixer (MTH-100, MIULAB). The digestion was terminated by adjusting the EDTA concentration to 20 mM. The digested DNA fragments were recovered using DNA purification beads (N411, Vazyme Biotech), and subjected to Illumina library preparation (N203-02, N204-02, Vazyme Biotech). The same tools and steps as for ATAC-seq were used when processing the MNase-seq data.

### Measurements of gene expression

To measure gene expressions, 0.2 g of each tissue was collected for RNA-seq. Two independent biological replicates were included. RNA isolation, library construction, and sequencing were performed by the Novogene Company (Beijing). Illumina NovaSeq 6000 was used for sequencing. The data were released with a yield rate higher than 95%. Raw reads were filtered by removing the adapter sequences and low-quality sequences with fastp v.0.20.1 (28). By using the Araport11 genome release of *A. thaliana* as a reference, the reads were then aligned to the genome with hisat2 v.2.2.1 (40). Next, samtools v.1.9 (41) was used to tidy the alignment results and remove the redundant sequences derived from PCR. After that, featureCounts v.2.0.3 (42) was used to process the alignment results for gene quantification. The read counts were normalized and represented with TPM (transcripts per million), by the following formula:


\begin{equation*}TPM\ = \ {10}^6\ *\ \frac{{read\ counts\ /\ gene\ length}}{{\mathop \sum \nolimits_{all\ genes} \left( {read\ counts\ /\ gene\ length} \right)}}\end{equation*}

To survey the distribution of sea-ATI CREs around *A. thaliana* genes, sea-ATI motifs were used to match the genomic sequences using the R package motifmatchr (31) with the *P*-value set to 0.0001. Visualizations were generated with the ComplexHeatmap R package (39).

### Dual-luciferase reporter assay

The reporter assay was performed to explore the transcriptional regulatory effects of sea-ATI motifs. First, the Arabidopsis protoplast was isolated from 4-week-old leaves as described in (43). Promoter sequences were designed to contain a mini 35S promoter, and in addition, 8 repeats of the consensus of the seaATId motifs (Supplementary Table 3). The synthetic promoters were cloned into the pGreenII 0800-LUC vector in front of the Firefly Luciferase (Fluc) reporter gene. The same plasmid also contained the Renilla Luciferase (Rluc) gene driven by the 35S promoter, which served as a reference to normalize for the transfection efficiency. The engineered plasmids were propagated in Top10 and extracted with the GoldHi EndoFree Plasmid Maxi Kit (CW2104M, CWBIO), and subsequently transfected into the isolated protoplasts mediated by PEG as described in (43). After transfection, the protoplasts were placed in the dark at room temperature for 10–16 h and then measured for luminescent strengths. Luminescence from both the Fluc and Rluc was measured by the kit (11402ES60, YEASEN) with a microplate reader (Spark, Tecan). The ratio between Fluc and Rluc is used to represent the promoter activity.

## Results

### Sea-ATI efficiently enriches sequences of active CREs

**Figure 1.**
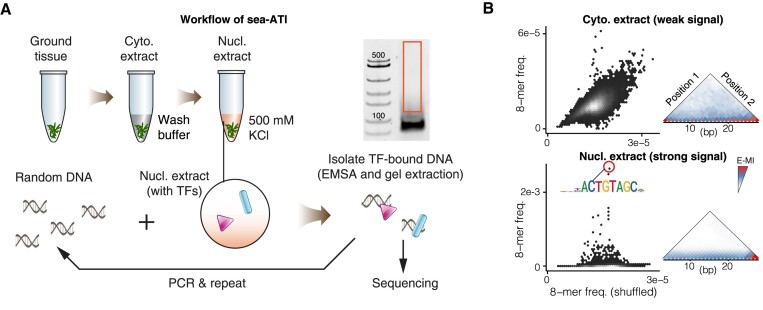
Sequential Extraction Assisted-Active TF Identification. (**A**) Schematic Representation of the Sea-ATI Workflow. Sea-ATI profiles the cistrome models of the active TFs by enriching ligands bound by TFs (in the nuclear extract) from a complex randomized DNA library. Ground powder of plant tissue is first extracted with a salt-free wash buffer to remove cytoplasmic components, then extracted with a high-salt buffer capable of lysing the nuclei to obtain nuclear proteins. After incubating the nuclear extract with the randomized DNA library, the protein-bound ligands are separated from the unbound ligands by EMSA and gel extraction, and PCR amplified. After 3–5 cycles of enrichment, the bound libraries are sequenced and analyzed. (**B**) The Sequential Extraction Enriches TFs by Removing the Cytosol. Left, log frequencies of all 8-mers are compared between the stem sea-ATI library and the same library shuffled. Right, E-MI analyses (see Methods); signal strength near the bottom of the triangle (hypotenuse) becomes stronger than elsewhere if TF signals are present. Note that TF signals are detected only in the sea-ATI library enriched with the nuclear extract (bottom) but not with the cytoplasmic extract (top). The corresponding motif of an enriched 8-mer (red circle) is indicated.

We first adapted the workflow of ATI (22) to allow low-input and facile characterization of plant samples. Nuclei isolation is a common first step for studying transcriptional control, but the yield is low (∼5–10%) for plant tissues. Grams of input materials are typically required for plant protocols with nuclei isolation (5,44,45). The workflow of sea-ATI (Figure 1A) bypasses nuclei isolation and reduces the input to ∼0.05 g. Specifically, the powder of ground tissues is first washed with chilled low salt buffer (0 mM KCl) to extract or remove cytoplasmic components, then a high salt buffer (500 mM KCl) is applied to extract nuclear proteins that contain the TFs (Figure 1A). Next, the nuclear extract is incubated with a complex library of 76-bp synthetic DNA ligands that contain a 30-bp randomized region. The ligands bound by TFs in the nuclear extract are then separated from the unbound ligands by the electrophoretic mobility shift assay (EMSA), eluted from the gel, and PCR amplified. The workflow is repeated for 3–5 rounds. After that, the DNA library is sequenced and analyzed for DNA-binding specificities to illustrate the landscape of the active cistrome. As expected, analyses show that most TFs preferentially locate in the nucleus (Figure 1B). Both the enrichment of DNA subsequences and E-MI (enriched-sequence-based mutual information) (33) indicate that the sequence selectivity of cytoplasmic proteins is low (Figure 1B, top), whereas proteins in the nuclear extract prefer to bind to highly specific DNA sequences (Figure 1B, bottom).

### Sea-ATI identifies novel specificities of plant active cistrome

**Figure 2.**
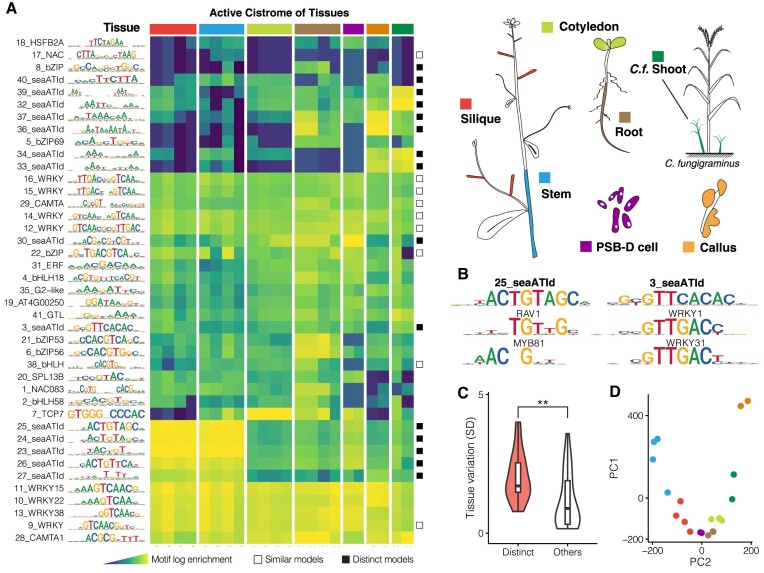
Sea-ATI Unveils Novel Specificities of Plant Active Cistrome. (**A**) The Active Cistrome of Different Tissues. Tissues examined with sea-ATI are illustrated (right) and indicated on top of the heat plot. Motifs are named according to the most similar reported motif. Numeric prefixes (1–-41) in the motif name serve as unique IDs. The motif models are classified into distinct models (▪), similar models (□), and reported models (no label) dependent on their similarity to previously reported plant motifs. (**B**) Distinct Model Examples. Distinct motif models align poorly with previously reported motifs (the two best alignments are shown below) and represent novel specificities of the active cistrome. See Supplementary Figure 1 for alignments of all distinct models. (**C**) The Activity of Distinct Models is Tissue-specific. Standard deviations of motif enrichment in the tissues are compared between distinct and other models (*t*-test, *P* = 0.006). (**D**) PCA of Sea-ATI Libraries. Gapped 10-mers spaced 0–8 bp in the middle were ranked according to enrichment. The ranks of the top 10 000 10-mers of each library were used for PCA.

We next applied sea-ATI to profile the active cistrome for seven different tissues: silique, stem, callus, PSB-D cell, root, cotyledon of *A. thaliana*, and shoot (young tiller) of *Cenchrus fungigraminus*, a C4 monocot with high photosynthetic efficiency and versatile applications (46). In total, 41 representative motif models (Figure 2A; Supplementary Table 2) were discovered *de novo* from these sea-ATI libraries. Each sea-ATI motif is assigned an ID (from 1 to 41)as the unique identifier. By comparing with 1618 non-redundant, previously curated *A. thaliana* motifs (see Methods), the sea-ATI motifs were classified into distinct (seaATId motifs), similar, and reported models that respectively share low, medium, and high similarity with the reported motifs (Figure 2A; Supplementary Figure 1). Unexpectedly, 36% (15 out of 41, Figure 2A) of the motifs are distinct models that align poorly with all reported motifs (Figure 2B), representing novel specificities of the active cistrome. This is in sharp contrast with the animal ATI results, wherein most of the motifs have been previously identified (22). Remarkably, the specificity of 23–26_seaATIds is highly similar to the ALOG family TFs examined in another ongoing work (47), indicating the biological relevance of the sea-ATI motifs.

The identified motifs show considerable variations in their tissue distribution and activity (Figure 2A). The WRKY and CAMTA motifs (IDs 9–16, 28, 29) are highly and pervasively active across all examined tissues. In contrast, 8_bZIP, 17_NAC, and 18_HSFB2A are weakly active in specific tissues. The activity of 7_TCP7 is prominent in cotyledon samples, wherein TCPs were known to suppress the formation of trichomes (48). The bZIP and bHLH TFs (IDs 5, 8, 38, 2, 4, 21, 6) tend to function in the root, corroborating their reported roles in root development (49,50). The distinct models can be highly active, but are more tissue-specific compared to reported and similar models (Figure 2C). This fact suggests that CREs of the distinct models might be responsible for cell lineage and tissue identities. For instance, consistent with the floral regulatory roles of the potential recognizer ALOGs (47), motifs of IDs 23–27 are highly active in silique and stem. Motifs of IDs 32–34 and 39 are among the most active CRE models in *C.f*. shoot samples. Motifs of IDs 36, 37 are particularly active in callus. In addition to the curated motifs, enrichment analysis can also be performed for sea-ATI libraries with all reported plant motifs. This identifies CREs with relatively weaker activities. For example, CREs of 118 reported motifs that are dissimilar to the 41 curated sea-ATI motifs are also active in root, including VND6 and FAR1 (Supplementary Figure 1E). As the motif models represent only a restricted space of sequence configuration, we also compared all sea-ATI libraries based on the ranks of the enriched gapped 10-mers (Figure 2D). The enriched sequences in callus are distinct from all other tissues, potentially because callus is less differentiated and experiencing cell-fate reprogramming.

### Thermodynamics and drifts in the evolution of dimeric WRKY CREs

**Figure 3.**
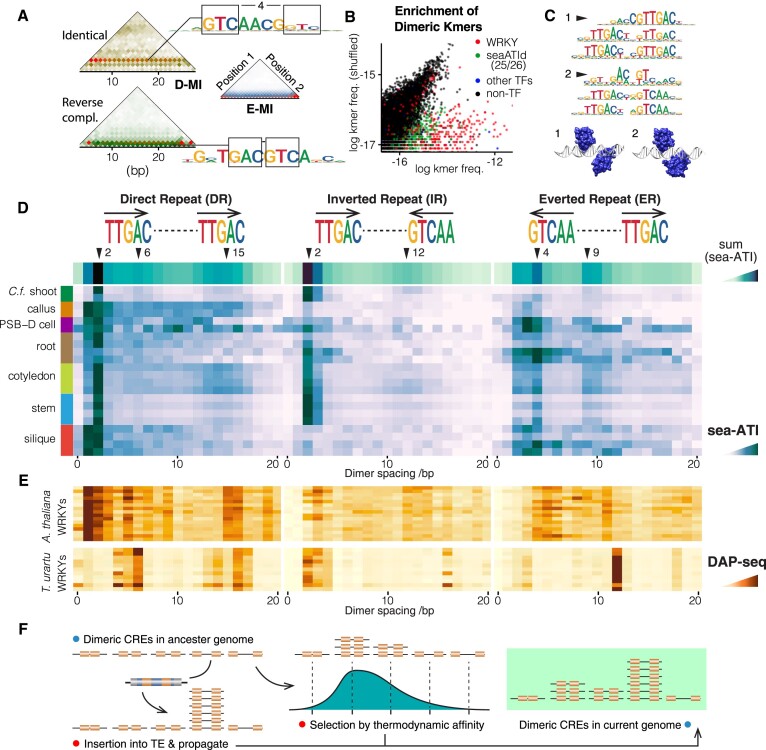
Dimeric WRKY CREs are Subject to Thermodynamic Selections and Evolutionary Drifts. (**A**) Detection of Universal Dimeric Bindings. D-MI analyses (left, see Methods) capture generic signals of dimeric TF bindings in the stem sea-ATI library, by measuring mutual information only from pairs of 3-mers that are identical (left, top) or reverse-complement (left, bottom). In comparison, E-MI (right) illustrates all strong modes of TF binding. The boxed pairs of positions on the enriched motifs have contributed to the indicated lines in the D-MI plots. (**B**) A Rich Diversity of Active Dimeric WRKY CREs. Log frequencies of gapped dimeric 10-mers (consisting of two identical or reverse-complement pentamers) are compared between the stem library and the same library shuffled. Points are colored according to the motifs that best match each 10-mer. Note that most 10-mers enriched by sea-ATI (points close to the x-axis) are colored, suggesting that they correspond to sea-ATI motifs. In contrast to WRKY (red) and the two seaATId TFs (IDs 25, 26; green), dimeric signals of other TFs (blue) can be highly enriched but the corresponding points are rare because they consist of monomeric bindings with a fixed orientation and spacing. (**C**) Specificity Change of Closely Spaced WRKY Dimers. Motif models of closely spaced WRKY dimers. Note the considerable specificity change (arrowheads) when spacing of the W-box cores (TTGAC) approach 1–2 bp. However, AtWRKY2 DBDs (6J4F) aligned according to the spacings lack direct protein-protein contacts (see Methods). (**D**) The Dimeric Binding Landscape of WRKY in Sea-ATI. Enrichment of dimeric WRKY CREs with different spacings and three relative orientations are shown for sea-ATI libraries. The row at the top sums up signals from all libraries to give an overview of the thermodynamic affinity landscape of WRKY dimers. (**E**) Species-Specific Configurations of Dimeric WRKY CREs. Enrichment of dimeric WRKY CREs with different spacings and three relative orientations are surveyed for DAP-seq peaks of *A. thaliana* (top) and *T. urartu* (bottom). Note that they both reveal binding preferences according to the affinity landscape from sea-ATI (shown in D), but with random drifts leading to inter-species discrepancies. Data (panel top to bottom): DAP-seq libraries for AtWRKY14, 18, 20, 24, 25, 27, 28, 29, 33, 3, 45, 50, 55, 65, 71, 75 (11); TuWRKY 10, 11, 12, 13, 15, 16, 19, 20, 21, 5, 8 (13). (**F**) Schematic of Dimeric WRKY CRE Evolution. Two forces are driving the evolution of dimeric WRKY CREs in the genome. First, CREs are randomly propagated by genetic drifting events such as TE amplification, this can occur even before the emergence of the cognate TF. Second, the population of dimeric CREs is refined based on thermodynamic affinity, retaining sites that are active to bind the TF and regulate transcription.

By dimerization, a limited set of TFs can recognize CREs with richer diversity (51,52) and enhanced selectivity (53,54). To provide an overview of generic signals from dimeric CREs, the summation of mutual information is taken over pairs of identical or reverse-complement sequences (D-MI; dimeric-sequence-based mutual information; see Methods). D-MI analyses (Figure 3A) indicate that sea-ATI libraries are enriched with signals of active dimeric CREs. Consistently, *de novo* motif discovery has identified dimeric motifs for TFs of both the reported models (e.g. bHLH, bZIP, NAC, WRKY, Figure 2A) and the distinct models (e.g. 30_seaATId and 39_seaATId, Figure 2A). For most TFs, the preferred dimeric binding consists of two monomers with a fixed orientation and spacing, resulting in enrichment of only a few gapped 10-mers (Figure 3B, blue points). However, dimers of WRKY and two seaATId TFs (IDs 25, 26) are more tolerant to variations in both parameters, leading to the enrichment of numerous gapped 10-mers (Figure 3B). The enrichment of WRKY is about 20-fold stronger than that of the seaATId TFs. Altogether, we discovered 45 dimeric WRKY motifs (Supplementary Figure 2A) with all possible relative orientations of the monomers, and observed between them a continuous span of the spacings. To reduce redundancy, only those with the highest activities are included in the curated set of models (Figure 2A). The binding specificity of WRKY can change drastically when monomers bind closely to each other in direct repeat (DR) or inverted repeat (IR) configurations (Figure 3C). We systematically profiled the sea-ATI libraries for orientational and spacing preferences of WRKY dimers (Figure 3D). The results show that the most favorable spacings are 1–4 bp, while large spacings of >10 bp are also observed. The orientational preference depends on the tissue; for example, the IR dimeric modes are active in stem and *C.f*. shoot but weak in silique and callus (Figure 3D). Direct structural contacts between DNA-binding domains (DBDs) are unlikely to occur for WRKY dimers (Figure 3C; Supplementary Figure 2B). However, WRKY can dimerize also through domain-swapping (55) and recognizes CREs on two parallel DNA strands. Whether CREs on the same strand (as observed here) are recognizable by domain-swapping requires further structural studies.

The dimeric CRE signals revealed by sea-ATI are developed from unbiased random sequences (Supplementary Figure 2C) and with sufficient incubation (22) to allow equilibrium. Consequently, the observed orientational and spacing preferences (Figure 3D) provide thermodynamic insights into biochemical affinities. Notably, the dimeric preferences of WRKY in monocot (*C.f*. shoot) and dicot (*A. thaliana*) samples are similar in sea-ATI (Figure 3D). In contrast, for WRKY DAP-seq binding assays that use *A. thaliana* and *T. urartu* genomic DNA (gDNA) instead of random sequences, considerable inter-species discrepancies are observed regarding the dimeric preferences (Figure 3E). The preferences also differ when using *A. thaliana* WRKYs to bind *A. thaliana* gDNA and random DNA (Figure 3D, E). As WRKYs do not change their intrinsic thermodynamic preferences in the assays, the observed discrepancies likely originate from the randomness of the genomic sequences — the evolutionary drifts. This means that dimeric WRKY CREs in the genome can be selected or propagated by driving forces other than their biochemical affinity. A major source of such driving forces in plants is the rapid expansion of transposable elements (TEs) under stresses (56). Indeed, in *T. urartu*, TEs that overlap with DNase I hypersensitive sites (DHSs) are rich in WRKY sites (13). The higher TE content in *T. urartu* (81.4% versus 18% in *A. thaliana*) could have more intensively expanded dimeric WRKY CREs with specific configurations, leading to a more discrete distribution of the spacing preferences (Figure 3E bottom versus top). Despite the evolutionary drifts, dimeric preferences of WRKY CREs of both *A. thaliana* and *T. urartu* still resemble the thermodynamic optima (Figure 3D, E). For example, the three preferred spacings of the DR configuration are 2, 6, 15 bp in sea-ATI; correspondingly, in genomic CREs, spacings of 1, 5, 15 are preferred in *A. thaliana*, and 6, 16 are preferred in *T. urartu*. Taken together, during evolution, thermodynamic selections and random drifts cooperated in shaping the WRKY CRE vocabulary of the plant genome (Figure 3F).

### The active cistrome is bound and affect chromatin accessibility

**Figure 4.**
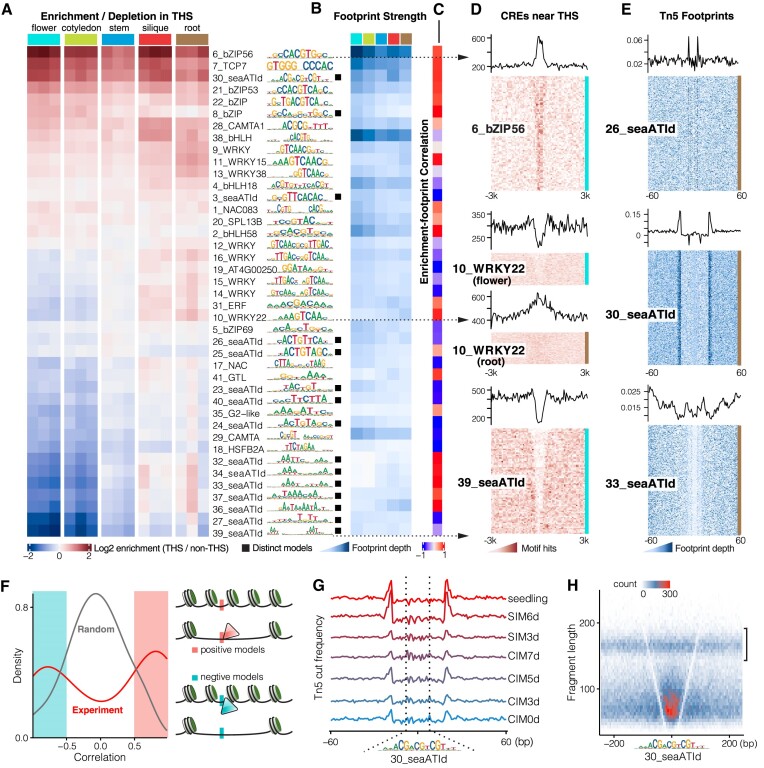
Chromatin Accessibility and Footprints Around the Active CREs. (**A**) Enrichment of Active CREs in Open Chromatin. The open chromatin, THSs (Tn5 transposase hypersensitive sites), are defined by ATAC-seq peaks of the *A. thaliana* tissues. The heatmap illustrates the density ratio of motif hits inside and outside of the THSs. Three replicates are shown for each tissue. (**B**) Strengths of Tn5 Footprints around the Active CREs. The strength of Tn5 footprints reveals occupancy of the CREs of each sea-ATI motif by cognate TFs. (**C**) Correlation between Open-chromatin Enrichment and Footprint Strength. The Pearson Correlation Coefficients are calculated based on data in (A) and (B) for each motif. (**D**) Distribution of Motif Hits around THSs. Each row in the heatmap represents a 6-kb region centered on a THS. The line plot on the top is the sum of motif hits at each position of the THSs. Color bars to the right of the heatmap denote the tissues according to (A). Note that biochemically active CREs can either enrich (6_bZIP56) or deplete (39_seaATId) in the THSs. (**E**) Tn5 Footprints at CREs of Distinct Models. Each row in the heatmap represents a 121-bp region centered on a CRE of the distinct motif models. The line plot on the top is the average footprint depth at each position of the 121-bp region. Note that footprint sizes can be larger than the lengths of the motifs because, for the cognate TFs, amino acids not bound to DNA also serve as steric hindrances to Tn5. (**F**) Bimodal Distribution of the Correlation Coefficients. Pearson Correlation Coefficients in (C) are either positive or negative (red line), whereas the bimodal distribution is not observed for randomly reordered data (black line). Motif models with correlation coefficients larger than 0.5 were defined as ‘positive models’, for which higher TF occupancy at the CREs is associated with higher nearby chromatin openness. Contrarily, the ‘negative models’ are those with coefficients smaller than -0.5, and occupancy of CREs thereof is associated with lower chromatin accessibility in the vicinity. (**G**) Footprints at Distinct Model CREs Vary in Regeneration. Hypocotyls of A. thaliana were cultivated on callus-inducing medium (CIM) and shoot-inducing medium (SIM) for different days and subjected to ATAC-seq (18). The central region demarcated by the dotted lines represents the width of the distinct motif 30_seaATId. (**H**) Short MNase-seq Fragments Overlap Distinct Model CREs. Counts of MNase-seq fragments (stem) are binned into 5 × 5-bp bins according to their lengths and center positions. Note that most fragments overlapping the 30_seaATId CREs (between the ‘V’-shaped footprint lines) are of sub-nucleosomal sizes (nucleosomal fragments indicated with bracket), corroborating that the CREs are occupied by TFs.

Accessible chromatin regions are known to enrich the binding sites of TFs and play a pivotal role in transcriptional regulation (3). To investigate whether the active CREs identified by sea-ATI are bound and how they associate with chromatin accessibility, we generated ATAC-seq libraries for flower, cotyledon, stem, silique, and root of *A. thaliana* (Supplementary Figure 3A). Tn5 transposase hypersensitive sites (THSs) were defined for the ATAC-seq assays to represent the open chromatin regions. To explore the relationship between sea-ATI CREs and chromatin accessibility, we initially examined the enrichment of CREs of all sea-ATI motifs in THSs (Figure 4A, D). Unexpectedly, only less than half of the motifs are consistently enriched in open chromatin across all tissues (Figure 4A, 6_bZIP56 exemplified in Figure 4D). Other motifs either distribute evenly inside and outside THSs, deplete in THSs (e.g. 39_seaATId in Figure 4D), or enrich/deplete depending on tissue identity (e.g.10_WRKY22 in Figure 4D). Note that highly active motifs detected in sea-ATI are not necessarily associated with the open chromatin. CREs of these motifs could have recruited nucleosomes upon binding. For example, the 23–26_seaATIds are highly active in silique and stem (Figure 2A) but are slightly depleted in THSs (Figure 4A). WRKY motifs (IDs 10, 14–16) are active in all sea-ATI samples, but their enrichment in THSs is tissue-dependent (Figure 4A, Figure 4D 10_WRKY22). The A/T rich motifs (IDs 32, 39) show activity in cotyledon, silique, and root (Figure 2A) but are depleted in THSs (Figure 4A, Figure 4D 39_seaATId) or delineate the border of the THSs (Supplementary Figure 3B). Although WRKYs can recognize genomic CREs corresponding to diverse dimeric configurations (Figure 3E), only the DR configurations spaced 1–3 bp are considerably enriched in the open chromatin (Supplementary Figure 2D). Overall, the reported and similar motif models show higher enrichments in THSs than the distinct models. The variable enrichment/depletion patterns of sea-ATI motifs suggest a complex relationship between CREs and chromatin accessibility.

When digesting chromatin with enzymes, CREs occupied by TFs are prevented from digestion, resulting in ‘footprint’ regions devoid of enzymatic cuts. Footprints were detected around CREs of all sea-ATI motifs (Figure 4B, E; Supplementary Figure 3C) including the distinct models (Figure 4E), providing unambiguous evidence that the newly identified cistrome models are bound by TFs. Motifs with stronger enrichment in THSs tend to exhibit stronger footprints (Figure 4A, B and Supplementary Figure 3D). To examine how TF occupancy at CREs of the sea-ATI motifs potentially affects local chromatin accessibility, we examined the correlation between footprint depth and chromatin openness (Figure 4C). The correlation coefficients of all sea-ATI motifs show a bimodal distribution (Figure 4F), suggesting that binding of TFs to most of the sea-ATI motifs either positively or negatively correlates with the local chromatin accessibility. Accordingly, we classified the sea-ATI motifs into ‘positive models’ and ‘negative models’. It is conceivable that CREs of the two model categories are recognized by TFs that contribute to chromatin openness in opposite ways. Consistent with this, the ‘positive models’ are more enriched in THSs than the ‘negative models’ (Supplementary Figure 3E). These findings are also consistent with prior observations that upon binding, TFs can either dissociate or stabilize the nucleosome (33). However, comparing nucleosome occupancies in the presence and absence of these CREs is required to further validate the observed correlations. Also, upon TF-binding to these CREs, the transcriptional influences can be complex because chromatin accessibility is not the sole determinant of transcription. In published ATAC-seq data series across *A. thaliana* shoot regeneration (18), footprints were also visible for the distinct models (Figure 4G; Supplementary Figure 3F) and varied in strength at different stages of regeneration. The footprint at 30_seaATId CREs (Figure 4G) is most prominent when explants were transferred to a shoot-inducing medium (SIM) for 6 days, implicating the potential role of the CREs in the formation of the shoot apical meristem. Fragments < 100 bp from MNase digestion offer an alternative approach to reveal the occupancy of regulatory proteins (20,57). We therefore generated MNase-seq libraries for stem (Supplementary Figure 3G), and observed the ‘V’-shaped footprint together with enrichment of subnucleosomal-sized fragments at 30_seaATId CREs (Figure 4H), further supporting their occupancy by TFs (57). By visualizing the coverage of >145 bp MNase-seq fragments, we also examined the distribution of nucleosomes around the CREs (Supplementary Figure 3H). Nucleosomes were found depleted at CREs of most sea-ATI motifs, presumably due to the binding of TFs. Collectively, these evidences indicate that the identified distinct models are recognized and bound, that is, they represent biochemically functional CREs.

### The active cistrome regulates transcription

**Figure 5.**
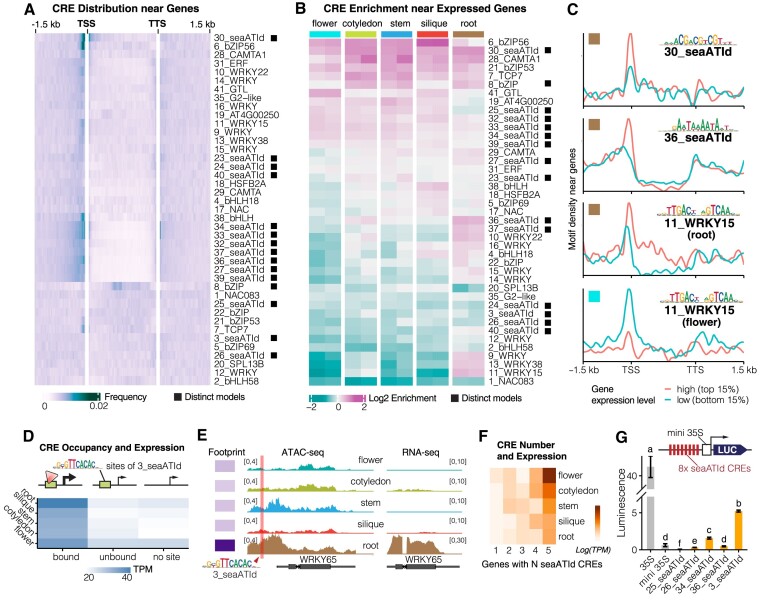
The Active CREs Enrich Around Promoters and Regulate Transcription. (**A**) Sea-ATI CREs Enrich Around Promoters. Distributions of the active CREs around *A. thaliana* genes were evaluated by the density of motif matches. Note that most CREs enrich strongly around TSS and weakly around TTS. The lengths of all transcripts were normalized to 2 kb. (**B**) CRE Enrichments Around TSS of Expressed Genes. For each motif, hits located ±300 bp around the TSSs were counted, and compared between genes with high (top 15%) and low (bottom 15%) expressions to derive the ratio of enrichment, which is then visualized in the heatmap. Two replicates are shown for each tissue. (**C**) CRE Distributions near Genes with Low and High Expressions. Examples of CRE distributions for individual sea-ATI motifs near genes with high (red) and low (blue) expressions; lines are LOESS smoothed with *n* = 500 and span = 0.1. (**D**) Occupied CREs are with Higher Activities. Median expression levels of genes associated with bound and unbound CREs of 3_seaATId, and of genes without a CRE site. The bound states were defined according to Tn5 footprints. (**E**) Occupancy of a 3_SeaATId CRE Correlates with Chromatin Accessibility and Expression. The CRE of 3_seaATId near WRKY65 (AT1G29280) has higher occupancy in root, consistent with the higher chromatin accessibility and expression level thereof. (**F**) Transcriptional Regulation by Distinct Model CREs is Accumulative. Genes harboring more CREs show higher expression levels (median values). Only bound CREs of the distinct models are considered. (**G**) Transcriptional Regulation by Distinct CREs. Consensuses of the seaATId motifs were repeated 8 times in front of a mini 35S promoter for the dual-luciferase reporter assay. Protoplasts from Arabidopsis leaves were used for transfection. Note that compared to mini 35S, adding seaATId CREs can either activate or repress transcription. Error bars show standard deviations. Groups labeled with different letters denote *P*< 0.05 in the *t*-test.

We next asked whether the active CREs identified by sea-ATI are involved in transcriptional regulation. Firstly, we examined the distribution of sea-ATI CREs around *A. thaliana* genes (Figure 5A). The majority of the CREs, including those of the distinct models, enrich strongly around the transcription start site (TSS) and moderately around the transcription termination site (TTS), with depletion observed in the gene body. These results agree with the previous finding that binding sites of *A. thaliana* TFs are typically located near the TSS, and that the peak of the distribution occurs at –50 bp (58). We also surveyed the TSS enrichments of all known motifs and found their extent of enrichment is similar to sea-ATI CREs (Supplementary Figure 4A). The moderate enrichment around the TTS suggests a potential role for the CREs in transcriptional termination. Notably, CREs of distinct model 8 enrich in the middle of the transcript (Figure 5A), indicating its possible involvement in the maintenance of transcription.

If the sea-ATI CREs activate or repress transcription when bound, their occurrence will enrich in promoters of genes with high/low expressions. We therefore measured expression levels by RNA-seq for tissues of *A. thaliana* (Supplementary Figure 4B). Comparing motif matches in promoters of highly expressed (top 15%) and silenced (bottom 15%) genes, we found that approximately 1/3 of the sea-ATI motifs consistently enriched in promoters of high-expression genes regardless of the tissue identity (Figure 5B). The expression-associated motifs include, for example, the bZIP motifs of IDs 6, 21, 8, 28_CAMTA1 and 7_TCP7. Half of the distinct models also fall into this category, including IDs 8, 30 (Figure 5B, C 30_seaATId), 25, 32–34, and 39. In contrast, silence-associated motifs such as 20_SPL13B and 1_NAC083 have their CREs consistently enriched in promoters of low-expression genes (Figure 5B). This is in line with the observations that some NAC and SPL family TFs are capable of repressing gene expression (59,60). CREs of the distinct models (IDs 24, 3, 26, 40) are also weakly associated with silence. The other motifs associate with expression in a tissue-dependent manner. For example, CREs of the distinct model 36_seaATId prominently associate with expressed genes in root (Figure 5C 36_seaATId), but not in other tissues (Figure 5B). It is noteworthy that WRKY CREs (especially for IDs 9, 11, 13) are clear positive predictors of expression in root, and at the same time clear negative predictors of expression in other tissues (Figure 5B, C 11_WRKY15), although WRKY CREs are biochemically active in all tissues (Figure 2A). These observations suggest that the CRE-expression relationship can be diverse, and that the tissue-specific context should be considered when assessing and modeling the effects of CREs. In root and silique, more sea-ATI CREs are associated with expressed genes than in other tissues (Figure 5B). Consistently, sea-ATI CREs in root and silique are overall characterized by higher chromatin accessibility (Figure 4A). Motif scores of the CREs only weakly correlate with expression (Supplementary Figure 4C, Pearson's *r* between –0.1 to 0.1), likely because CREs are defined with stringent matches of the motifs (*P*-value cut-off 1e-4). Consequently, all the CREs provide sufficient affinity to the cognate TFs.

The Tn5 footprints at distinct model CREs indicated their occupancy by TFs (Figure 4E). According to the footprint depth, TOBIAS (38) can further define bound and unbound CRE sites for a target motif (Supplementary Figure 4D). We found that genes near bound CREs of the 3_seaATId motif are characterized by higher expression levels compared to genes near the unbound CREs (Figure 5D). Moreover, the expression levels of the unbound genes are similar to genes without a 3_seaATId CRE. These results suggest that only the TF-bound CREs of 3_seaATId are active in transcriptional regulation, and that 3_seaATId CREs are recognized by transcriptional activator TFs despite their weak association with silenced genes (Figure 5B, Supplementary Figure 4E). For example, the 3_seaATId CRE located 320-bp upstream of the TSS of WRKY65 (AT1G29280) has the highest occupancy in root than in other tissues (Figure 5E). Accordingly, the chromatin accessibility near the 3_seaATId CRE is highest in root, where the expression level of WRKY65 is also the highest (Figure 5E).

ChIP-seq datasets reveal highly confident CRE regions *in vivo*. Because CREs tend to cluster in the genome, peaks in a single ChIP-seq assay can enrich motifs from TFs other than the IP target (61). Accordingly, we explored multiple seedling ChIP-seq datasets and found that about 2/3 of the sea-ATI motifs are enriched in ChIP-seq peaks (Supplementary Figure 4F). Most prominent is that the distinct model 30_seaATId strongly enriched in all eight datasets. Next, we examined the number of functional CREs in *cis*-regulatory regions, as the number was found to positively correlate with gene expression (62). In line with this previous evidence, the results suggest that when a promoter contains more bound CREs of reported or similar sea-ATI models, the expression of the regulated gene is higher (Supplementary Figure 4G). This is also true for bound CREs of the distinct models (Figure 5F), further supporting that the distinct models represent functional CREs from the aspect of transcriptional regulation.

Finally, to experimentally explore how the distinct CREs regulate transcription, we performed reporter assays and found that these CREs can either activate (Figure 5G, 3, 34_seaATIds) or repress transcription (Figure 5G, 25, 26_seaATIds). The observed transcriptional activation by 3_seaATId is consistent with the higher expression near occupied 3_seaATId sites (Figure 5D).

### The active cistrome spans closed chromatin and silenced genes

**Figure 6.**
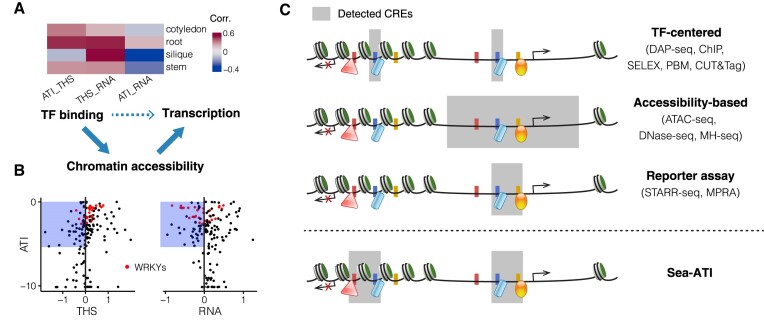
Sea-ATI Reveals Active CREs in Closed Chromatin and near Silenced Genes. (**A**) Correlation between Features around Sea-ATI CREs. The binding activity of the CREs is measured by sea-ATI (data from Figure 2A). The chromatin accessibility near the CREs is measured by ATAC-seq (THS, data from Figure 4A). The transcription level of genes downstream of the CREs is measured by RNA-seq (data from Figure 5B). (**B**) Active CREs in Inactive Chromatin Regions. For the motifs of sea-ATI CREs, their biochemical activity is respectively plotted against their enrichments in open chromatin (left) and in active promoters (right). The blue-shaded areas indicate highly active CREs (activity > 29% of the strongest motif) that enrich in closed chromatin or near the silenced genes. (**C**) Methods for Plant CRE Discovery. Compared to other plant protocols, one of the key strengths of sea-ATI is that it can detect all CREs that are capable of binding to TFs, regardless of whether they positively or negatively regulate chromatin accessibility and transcription. MH-seq: MNase hypersensitivity sequencing (20).

By combined data analyses of sea-ATI, ATAC-seq, and RNA-seq for genomic sites of the sea-ATI CREs, we found that the TF-binding activity of the CREs positively associates with the nearby chromatin accessibility (Figure 6A, ATI_THS). The accessibility around sea-ATI CREs also facilitates the expression of the targeted genes (Figure 6A, THS_RNA). However, the correlation is not obvious between TF-binding at the CREs and the expression of the targeted genes (Figure 6A, ATI_RNA). The results suggest that TF binding is an important determinant of chromatin accessibility, which in turn determines the level of transcription. However, the influence of TF binding on transcription can be multidirectional and indirect (Figure 6A, bottom).

We next evaluated whether sea-ATI offers unique insights into plant active cistrome compared to other approaches. We examined the enrichment of all Arabidopsis motifs in CRE sequences from sea-ATI, THSs, and active promoters (Supplementary Figure 5), and found that in general THSs and active promoters enrich similar CREs. In contrast, the CREs enriched in sea-ATI libraries represent a more distinct set (Supplementary Figure 5A). Of note is that sea-ATI has identified biochemically active CREs that enrich in closed chromatin or promoters of silenced genes (Figure 6B, dots in blue-shaded region; Supplementary Figure 5B), such as CREs of the WRKY TFs (Figure 6B, red dots). The transcriptional silencing effect is also observed for CREs of the 25, 26_seaATIds (Figure 5G). The active CREs in inactive genomic regions are of particular interest because their systematic discovery for plant tissues is yet not realized by other protocols (Figure 6C, see also the Discussion).

## Discussion

Sessile plants exhibit remarkable phenotypic plasticity, allowing them to rapidly respond and adapt to environmental changes (63). Central to such plasticity is the rich dynamics of the gene regulatory network wired by the binding of TFs to CREs in transcriptional regulatory regions (64). The biochemically active CREs that can bind TFs are thus of particular importance. Although ATI has established a first-principle method for profiling the intact landscape of the active cistrome (22), it is yet to be applied to plant samples and is inefficient in our preliminary trials. In this study, we report an adapted workflow, sea-ATI, which requires only ∼0.05 g of plant tissue as input. Applying sea-ATI to seven different plant tissues from both dicot and monocot revealed their active cistrome, and unexpectedly, identified 15 previously unreported motif models. Further analyses confirmed that the new models represent functional CREs.

Including the original ATI, the significant material loss during nuclei isolation is a common challenge encountered when applying many epigenetic protocols to plant samples. Typically, these protocols involve nuclei isolation as the initial step to remove irrelevant cytosolic ingredients and facilitate subsequent chemical or enzymatic reactions (5,44,45,65–67). The nuclei isolation step includes filtration (68,69), whereby the presence of the cell wall traps large amounts of nuclei in the debris that fails to pass the meshes. This results in a loss of over 90% of the materials. To improve efficiency, there were two main strategies: (i) isolating the protoplast and (ii) using the whole cellular mixture. It becomes facile to extract and recover intact nuclei after the enzymatic digestion that removes cell-wall components and releases the protoplasts (70,71), but protoplast isolation is laborious and invokes additional physiological responses (72). Alternatively, the whole cellular mixture has been utilized to skip nuclei isolation in protocols such as eChIP-seq (6). However, including cytosol in the reactions could increase the background or require more specific antibodies, especially in mature plant tissues like mesophyll or fruit flesh that are characterized by cells of very low nuclear-to-cytoplasmic ratios. In this work, the sequential extraction workflow in sea-ATI suggests another workaround to remove the cytosol while maintaining the maximum recovery of the nuclear contents. To separate the cytosolic ingredients from the nuclei, the ground tissue of plants is first washed or extracted with a low-salt buffer. Subsequently, the contents in the nucleus are released by extracting with high salt and detergent. In replacement of the second extraction, sonication or enzymatic digestion can also be applied to meet the requirements of other epigenetic protocols.

In complement to the existing toolkit for genome-wide discovery of plant CREs (4,73–75), sea-ATI identifies CREs purely based on their biochemical affinities to TFs. The unique detection scope of sea-ATI is not yet covered by available approaches for plants (Figure 6C). Specifically, TF-centered methods (e.g. DAP-seq, ChIP-seq) interrogate CREs of one TF at a time, while sea-ATI illustrates all active CREs. Measuring CRE activities for multiple TFs in one assay facilitates reliable comparisons. Additionally, sea-ATI offers single base-pair resolution and reveals also the CREs not located in the open chromatin, thus providing greater details than chromatin accessibility-based methods (e.g. ATAC-seq, DNase-seq). The cistrome detected by sea-ATI includes both activator and repressor CREs of transcription, this is in contrast to the paralleled reporter assays (e.g. STARR-seq, MPRA), which detect only CREs capable of activating transcription, and whose applications are currently limited to specific plant tissues such as leaf or protoplasts (24–27). Similar to SELEX, sea-ATI utilizes an input library containing 2–4 trillion randomized DNA ligands. The complexity is ∼670 000 times that of the Arabidopsis genome, which enables sea-ATI to derive CRE models that accurately describe the biochemical affinity of TFs, especially for high information-content models of the dimeric CREs (Figure 3D). By comparing CRE models derived from sea-ATI and genomic DNA-based methods (e.g. DAP-seq, ChIP-seq, ATAC-seq), the discrepancies can provide valuable insights into cistrome evolution (Figure 3F), chromatin structure, DNA modification, and interactions between TFs and other proteins. Motif discovery of sea-ATI starts from seed identification followed by multinomial model-based motif construction. The strategy enables the separation of closely related models (e.g. 24, 25_seaATIds). Moreover, sea-ATI does not require the use of antibodies, enzymes, or labeled recombinant proteins, making it straightforward to implement. Altogether, sea-ATI offers a comprehensive, high-resolution, yet simple and cost-effective approach for profiling the active cistrome in plant epigenetics research.

Recent technological and computational advancements have significantly enhanced the annotation of plant CREs (76–78), with a few efforts reaching the single-cell level (79,80). However, even in *A. thaliana* the catalog of CREs remains far from complete (4). This study systematically generated motifs of active CREs and found that 1/3 of them were previously unidentified, while another 1/3 showed discernable differences from reported models. The distinct and similar sea-ATI models represent functional CREs that influence chromatin accessibility and transcription (Figures 4 and 5). Many of these models exhibit high activity levels in tissues (Figure 2A), highlighting their potential physiological significance. These models not only reveal novel functional CREs helpful for future experimental designs, but also facilitate the mechanistic study of transcription by machine learning, whereby functional CREs serve as the fundamental predictors (81). At present, motif models are only available for approximately 1/3 of *A. thaliana* TFs (11) and are even more limited for other plant species. We believe that as time passes, TF-centered approaches will accumulate more data on the binding specificity of individual TFs and TF combinations, and ultimately decipher the origins of all *cis-*regulatory codes. Alternatively, for a specific CRE sequence of interest, the proximal biotinylation method followed by protein mass spectrometry can provide more abundant information (82). This approach not only identifies the TF that recognizes the CRE sequence but also reveals the cofactors that build up the regulatory machinery, thereby offering a deeper insight into transcriptional regulation.

CREs have emerged as promising next-generation breeding targets (83–86). Editing CREs not only allows for fine-tuning of gene expression to achieve the optimal dosage, but also helps eliminate detrimental side effects caused by gene pleiotropy (87). However, editing of CREs generally leads to downregulation of the target gene (84,85,88), partially due to the use of open chromatin regions and active histone markers in target selection. A key strength of sea-ATI is that the identified active CREs are not biased toward the positive regulatory elements. It is likely that some of the sea-ATI CREs enriched in closed chromatin or promoters of the silenced genes represent editing targets for the upregulation of gene expression.

In summary, sea-ATI offers a powerful tool for studying transcriptional regulation and epigenetics of plants. It allows profiling of the tissue-specific regulatory landscape and expands our understanding of the functional non-coding genome. The obtained CRE models serve as a valuable starting point for further investigations into the recognition, physiological function, and potential agronomic applications of the active cistrome.

## Supplementary Material

gkad853_Supplemental_Files

## Data Availability

All sequencing data have been deposited to China National Genomics Data Center under accession PRJCA017278.
