# Supplementary material for: Sea-ATI unravels novel vocabularies of plant active cistrome
Source: Nucleic Acids Res. 2023 Oct 18;51(21):11568–83. doi: 10.1093/nar/gkad853 (PMC10681729; doi:10.1093/nar/gkad853)
Supplement: gkad853_Supplemental_Files [file gkad853_supplemental_files.zip › Supplemental_figures_seaATI_20230711.pdf]

Sea-ATI Unravels Novel Vocabularies of Plant Active Cistrome

SUPPLEMENTAL FIGURES

Supplemental Figure 1

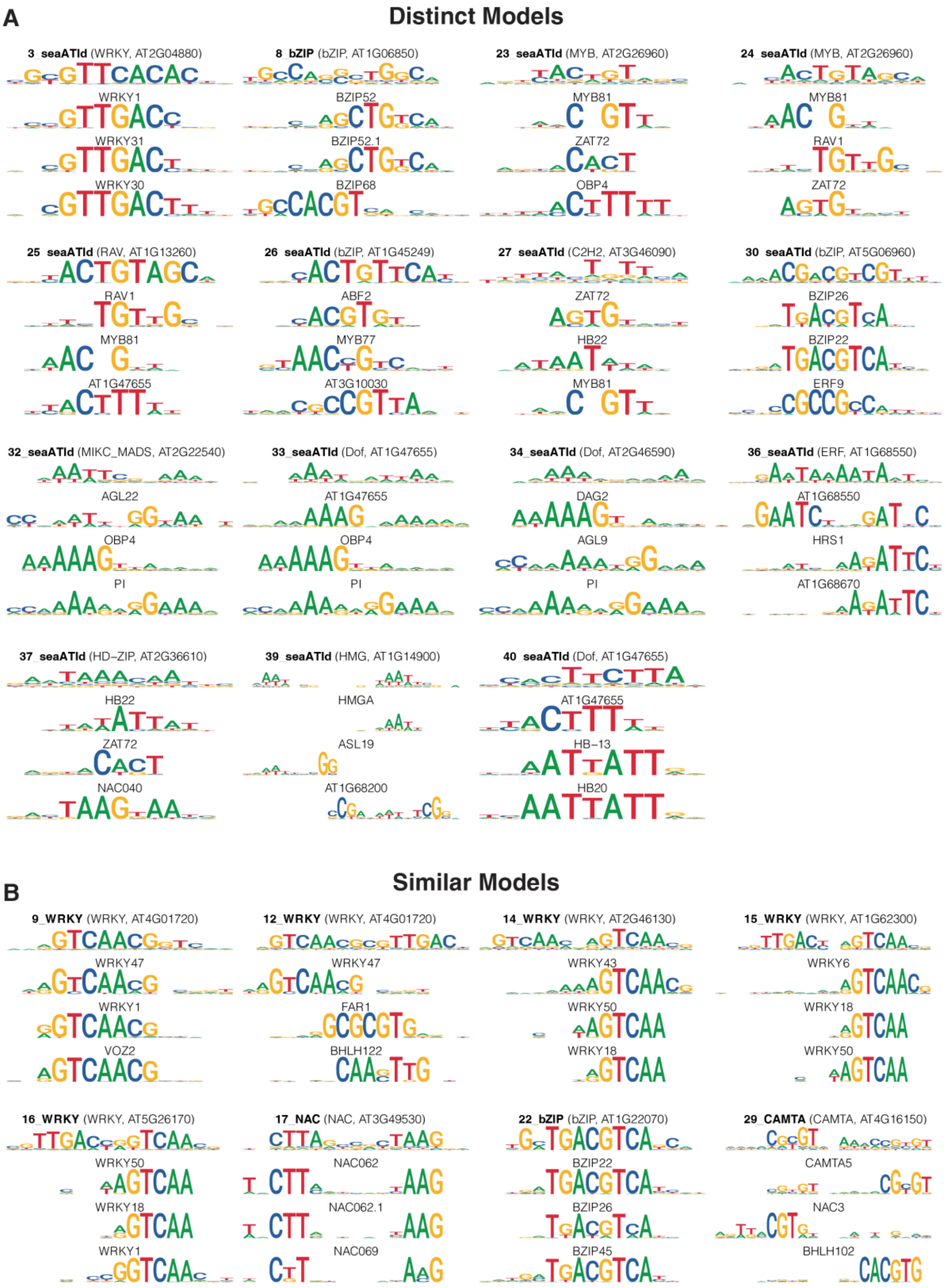

Supplemental Figure 1

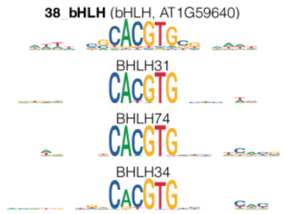

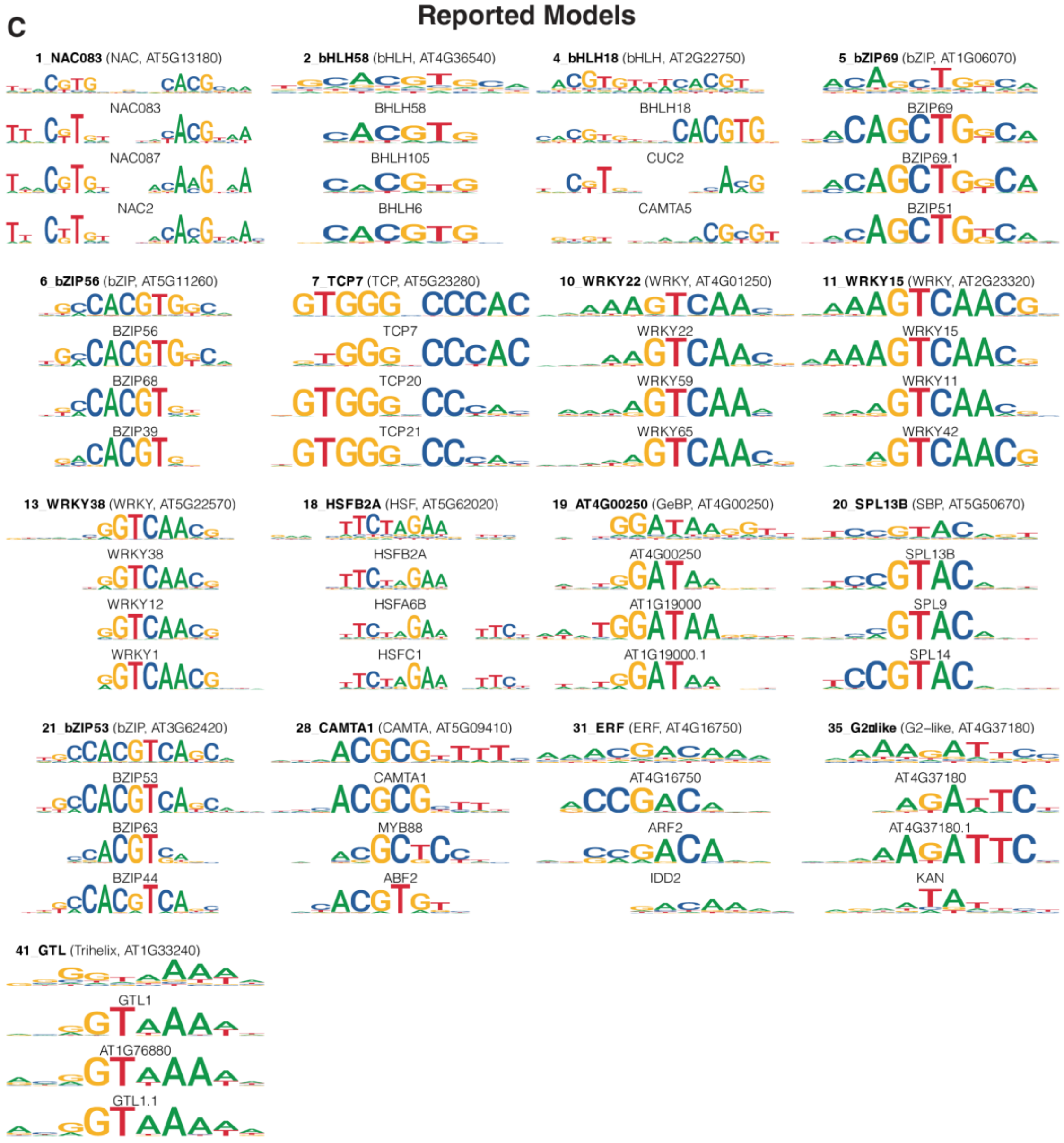

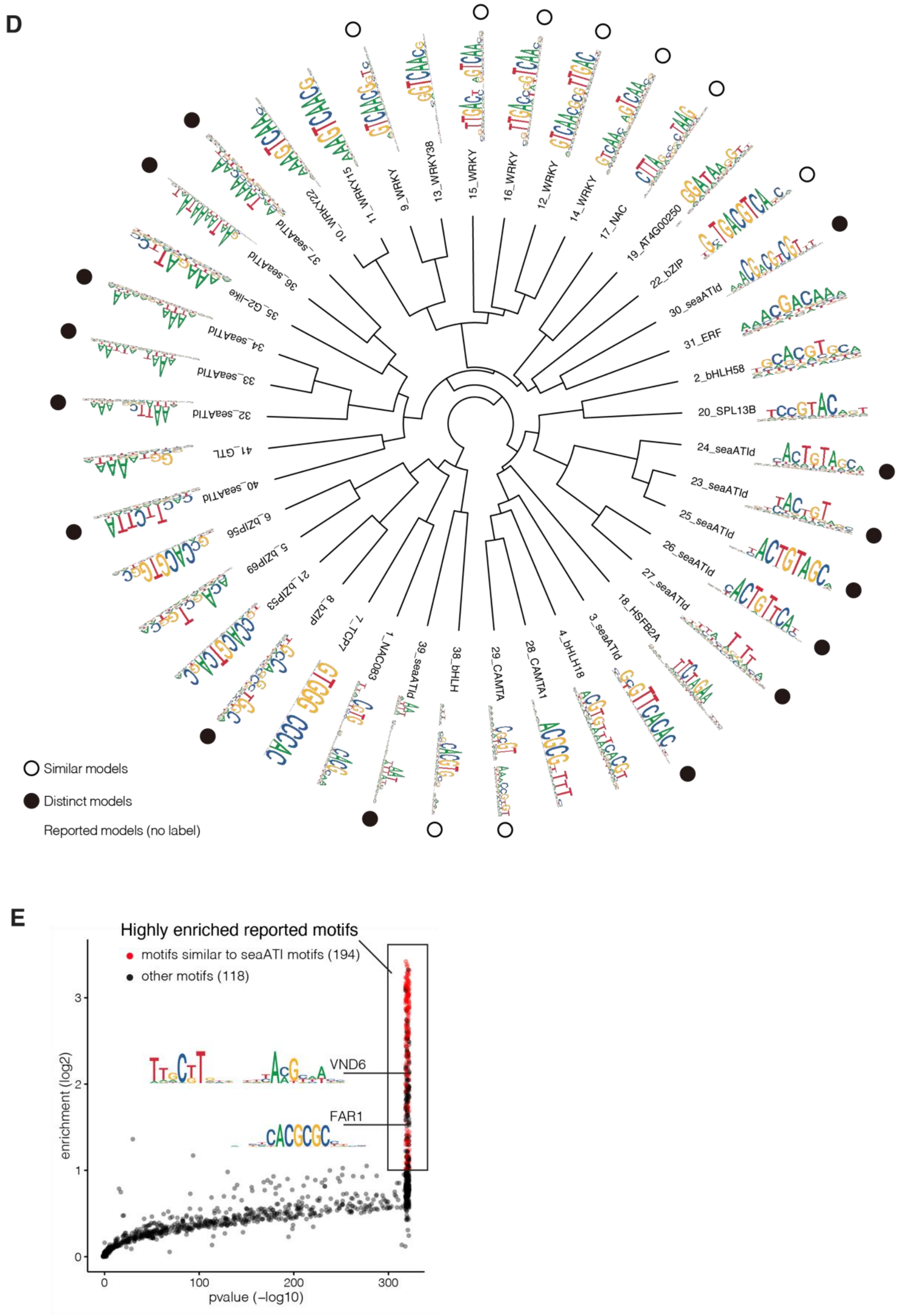

Supplemental Figure 1 | Three Categories of Motif Models Identified by Sea-ATI

(A–C) Classification of Sea-ATI Models into Three Categories. Based on the similarity between sea-ATI models and reported motifs of A. thaliana TFs, the sea-ATI motif models were classified into distinct, similar, and reported models that respectively share a low (**A**), medium (**B**), and high (**C**) similarity with previously reported motifs (see Methods).

Motifs are named according to the most similar reported motif, or as "seaATId" if similarity is low. Numbers (1–41) in the motif name serve as unique IDs.

(D) Hierarchical Clustering of the Motif Models Identified by Sea-ATI.

(E) Weaker Sea-ATI Signals Detected by Enrichment Analysis of Known Motifs. Reads in the sea-ATI libraries of seedling root are matched with 1618 reported motifs of Arabidopsis TFs (p=0.00001 for motif mapping). Highly confident enrichments (points in the rectangle, $-log_{10}(p)>300$ and $log_2(enrichment)>1$) of 312 motifs were detected. Note that 118 of the 312 motifs are considerably dissimilar to all curated sea-ATI motifs (black points), representing CREs that are active in root but not described by the *de novo* discovered motif models in (**D**). Two of these enriched motifs (VND6 and FAR1) are illustrated. Enrichment is calculated by comparing motif hits in root sea-ATI reads and in the shuffled reads. P-values are calculated assuming a Poisson distribution of motif occurrences on random DNA. A known motif is defined as dissimilar when its Pearson's correlation coefficient is smaller than 0.75 compared with all sea-ATI motifs.

Supplemental Figure 2

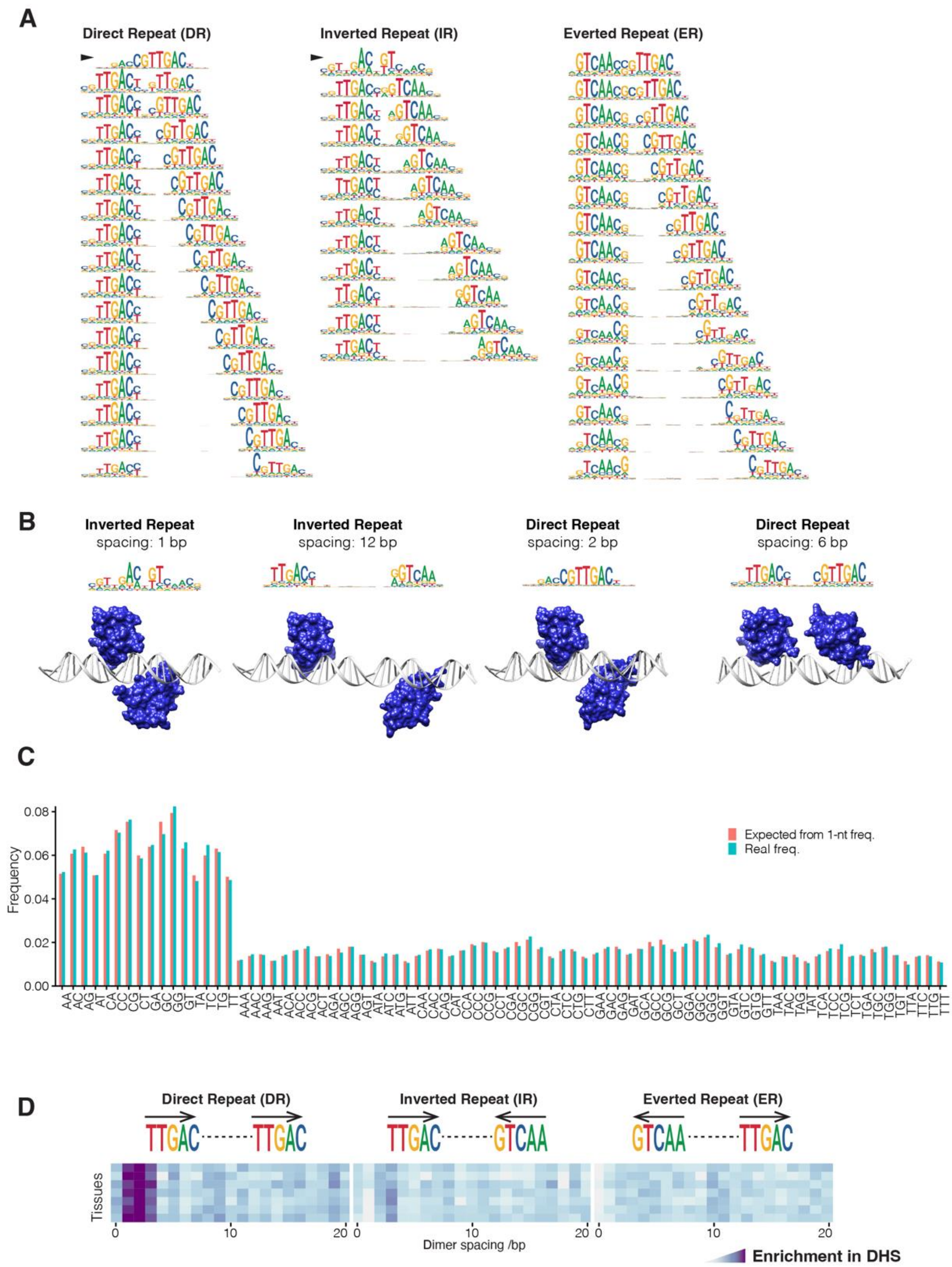

Supplemental Figure 2 | Dimeric Binding Modes of WRKYs

(A) The 45 De novo Motifs of WRKY Dimers from Sea-ATI Libraries. Arrows indicate motifs of closely spaced WRKY dimers where considerable specificity changes occur.

(B) Absence of Direct DBD Contact in Dimeric Binding of WRKYs. To mimic dimeric binding, the structure of the WRKY-DNA complex (PDB 6J4F) was aligned to B-DNA models (see Methods) with spacings and relative orientations observed in sea-ATI. Note that even the closely spaced dimers lack obvious contact between DBDs.

(C) The Input Library of Sea-ATI is Unbiased. For the input library, the actual di-nucleotide and tri-nucleotide frequencies are plotted aside of those expected by mono-nucleotide frequencies. The high accordance of the expected (red) and actual (blue) values supports the randomness of the input.

(D) Dimeric WRKY CREs in Open Chromatin. Enrichment of dimeric WRKY CREs with different spacings and three relative orientations are surveyed for DHSs of A. thaliana. Data (panel top to bottom): DNase-seq libraries for flower (1), flower (2), inflorescence (3), leaf (4), seedling (1), root (4), root (2).

Supplemental Figure 3

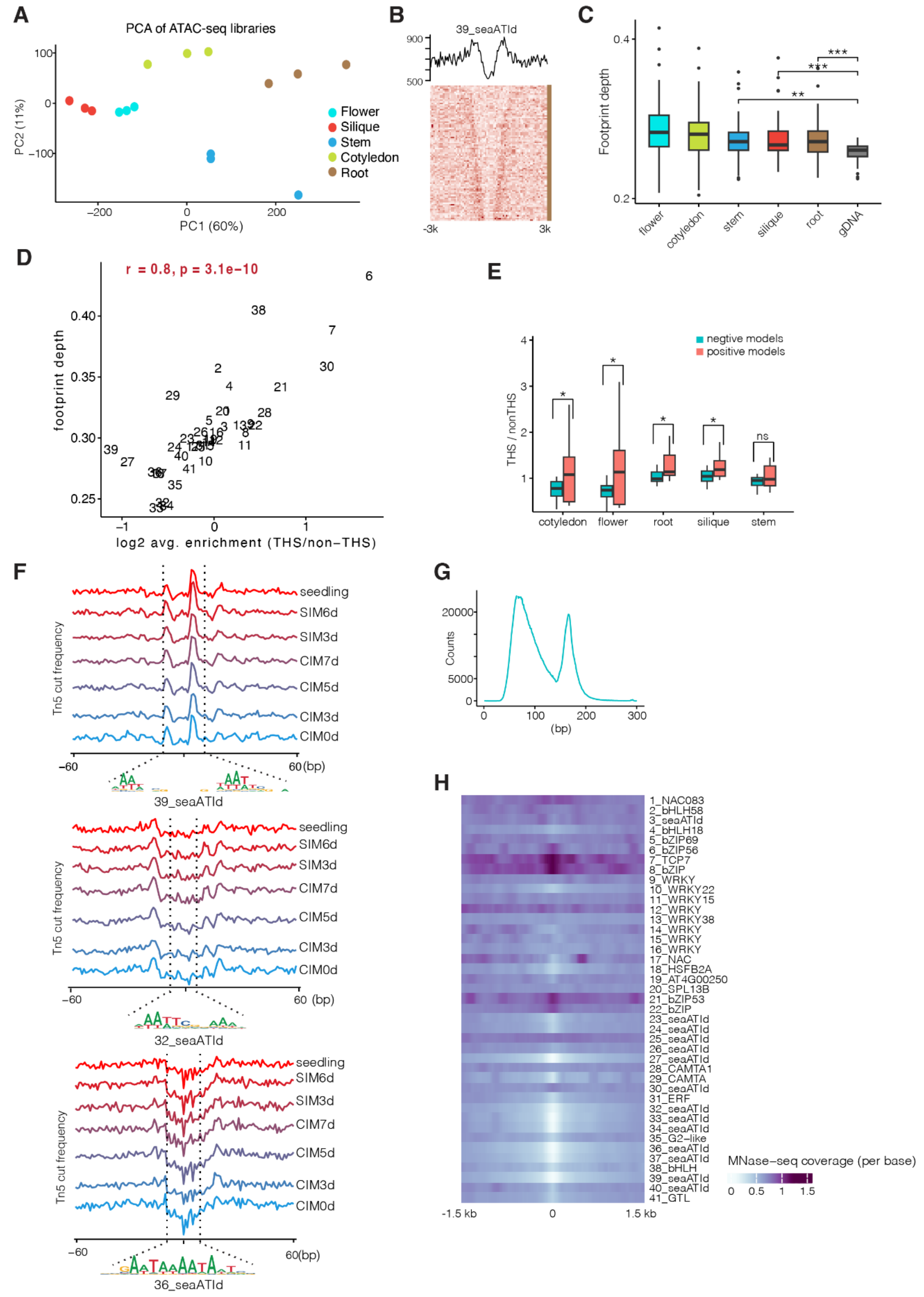

Supplemental Figure 3 | Chromatin Accessibility around the Sea-ATI CREs

(A) PCA of the ATAC-seq Libraries.

(B) Enrichment of CREs at THS Borders. CREs of 39_seaATId are surveyed for their distribution around THSs. Each row in the heatmap represents a 6-kb region centered on a THS. The line plot on the top is the sum of motif hits at each position of the THSs. Color bars to the right of the heatmap denote the tissue identity (root).

(C) Tn5 Footprints on Chromatin and Free DNA. Tn5 footprint depths in each tissue are compared to those on the protein-free genomic DNA. Note that stronger footprints are observed for the chromatin context in all tissues, suggesting that TF occupancy has increased the footprint depth.

(D) Motif Enrichment in THS Correlates with Footprint Depth. For each sea-ATI motif, the average enrichment in THS (across all tissues) is plotted against the average footprint depth. Motif ID is used as the symbol to represent each data point. Pearson's r and p-value for the correlation are also indicated.

(E) Higher Enrichment of Positive Model CREs in THSs. Motif enrichments in THSs were compared between "positive models" and "negative models" for tissues of A. thaliana. The boxplot shows medians (middle bar), quartiles (boxes), and 1.5 × interquartile ranges (whiskers). Note that "positive models" are generally more enriched in THSs. "*" indicates $p < 0.05$ (t-test).

(F) Footprints of Distinct Model CREs in Regeneration. Hypocotyls of A. thaliana were cultivated on callus-inducing medium (CIM) and shoot-inducing medium (SIM) for different days and subjected to ATAC-seq (5). The central region demarcated by the dotted lines represents the width of the presented motifs.

(G) Length Distribution of the MNase-seq Fragments. To study the occupancy of TFs at genomic CREs of the sea-ATI motifs, stem MNase-seq libraries with both nucleosomal- and subnucleosomal-sized fragments were generated.

(H) Nucleosome Distributions near Sea-ATI CREs. MNase fragments (stem) with lengths >145 bp and located in non-THS areas were selected and calculated for per-base coverage around the sea-ATI CREs.

Supplemental Figure 4

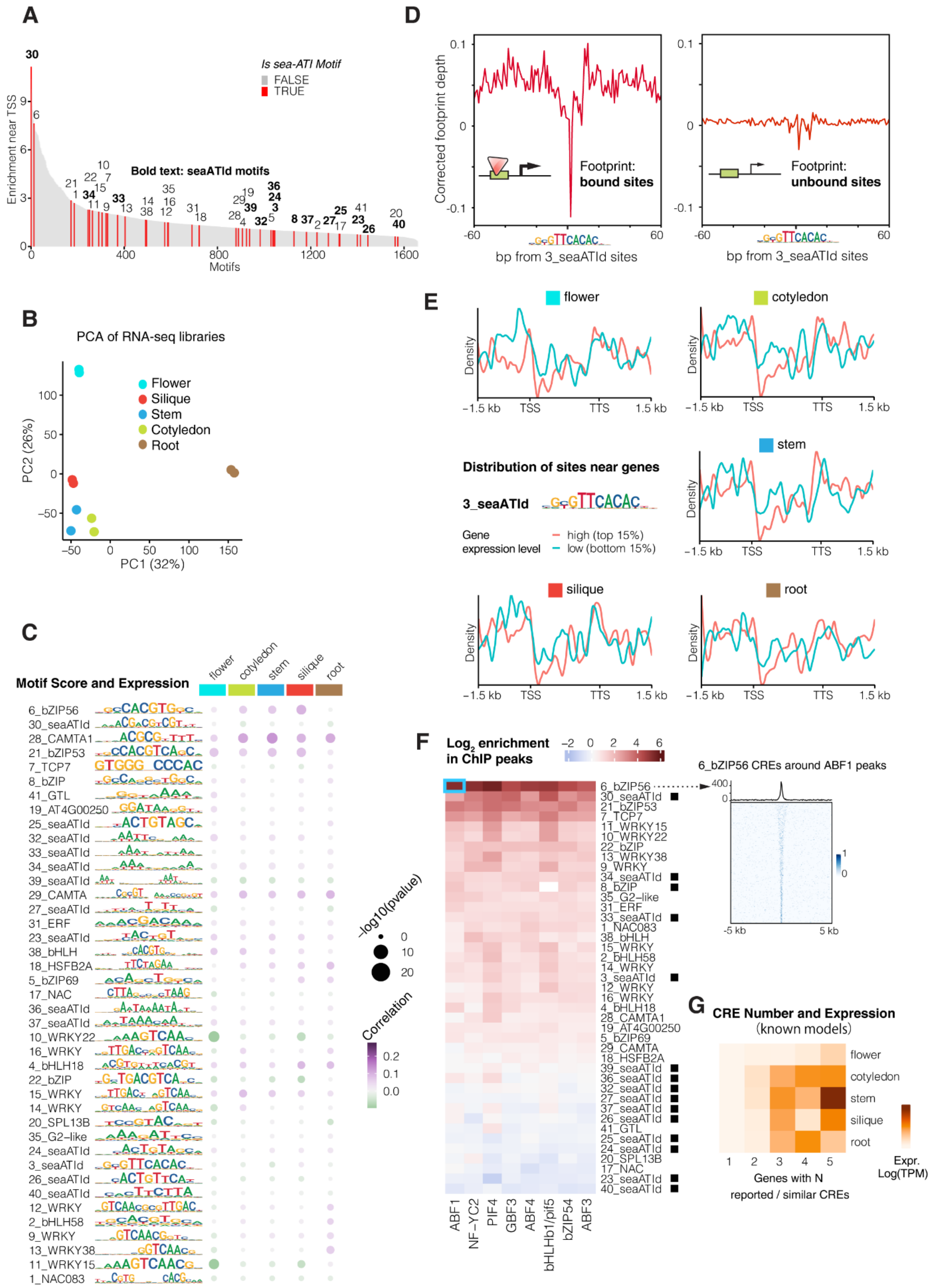

Supplemental Figure 4 | Transcriptional Regulation by Sea-ATI CREs

(A) **TSS Enrichments of All Motifs.** For each motif, hits located ±300 bp around the TSSs were counted and compared to the hits in the shuffled sequences to calculate the fold enrichment. IDs of sea-ATI motifs are labeled. Note that sea-ATI motifs (red) have a similar extent of enrichment compared to all reported motifs.

(B) **PCA of the RNA-seq Libraries**.

(C) **Correlation between Motif Score and Expression**. CREs are associated with motif-matching scores reflecting their binding affinities to the cognate TFs. The correlation between motif score and gene expression, together with the p-value of the correlation, are calculated and visualized for each motif and each tissue. Only CREs within 500 bp from TSSs of genes were considered in the analysis.

(D) **Footprints for Bound and Unbound Sites**. The CREs of 3_seaATId in the genome were classified into bound and unbound states according to the depth of Tn5 footprints in ATAC-seq.

(E) **CRE Distributions around Genes**. For 3_seaATId CREs, the distributions near genes with high (top 15%) and low (bottom 15%) expression levels were similar in all tissues; lines are LOESS smoothed with n = 500 and span = 0.1.

(F) **Enrichment of Sea-ATI CREs in ChIP Libraries**. Sequences in the peaks of 8 ChIP-seq libraries were examined for the enrichment of sea-ATI CREs, by comparing the matches of each sea-ATI motif in the peaks and in the shuffled peak sequences (p=1e-5 for motif matching). The enrichment of 6_bZIP56 in ABF1 ChIP-seq library (blue rectangle, left) is further illustrated for individual peak regions with the heatmap metaplot (right). Accession numbers of the ChIP libraries are GSM2130866, 2130888, 1665427, 2130872, 2130970, 1977462, 2130898, 2130976, and 2130934.

(G) **Transcriptional Regulation by Reported Model CREs is Accumulative.** Genes harboring more CREs show higher expression levels (median values). Only bound CREs of the reported and similar models are considered.

Supplemental Figure 5

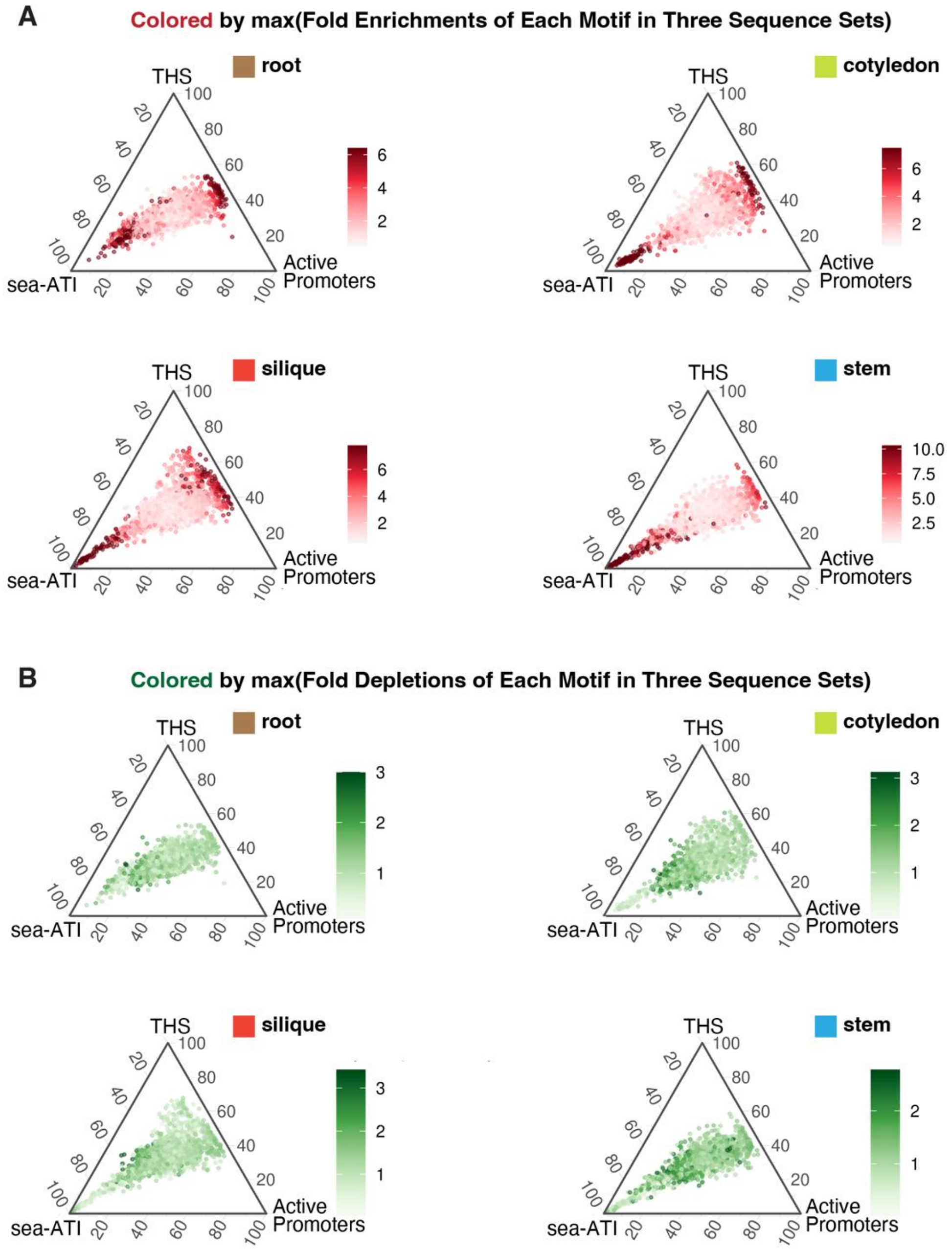

Supplemental Figure 5 | Sea-ATI Offers Distinct Information on Plant Cistrome

(A, B) Enrichments of all Available Motifs in Different Sets of Cistrome Sequences. Each dot represents a motif. While the ternary plots show relative enrichments between the sets, the maximum absolute enrichment is color-coded in **(A)**, and the maximum absolute depletion is color-coded in **(B)**. Note that motif enrichments are similar between THS and active promoters, whereas sea-ATI enriches a unique set of motifs, suggesting that sea-ATI complements THS and active promoter sequences for profiling the active cistrome. Also noteworthy is that motifs depleted in THSs and active promoters can have moderate enrichment in sea-ATI libraries (**B**), potentially because these motifs are biochemically active for binding but act as repressors.

The used cistrome sequences are sea-ATI libraries (Sea-ATI), peaks of ATAC-seq (THS), and -1000 bp to +500 bp around TSSs (Active Promoters). Sea-ATI motifs together with the 1618 reported Arabidopsis motifs were analyzed (p=0.00001 for motif mapping). Enrichment or depletion is calculated by comparing motif hits between the original and shuffled sequences.

References

1. Zhang,W., Zhang,T., Wu,Y. and Jiang,J. (2012) Genome-wide identification of regulatory DNA elements and protein-binding footprints using signatures of open chromatin in Arabidopsis. *Plant Cell*, **24**, 2719–2731.

2. Sullivan,A.M., Arsovski,A.A., Thompson,A., Sandstrom,R., Thurman,R.E., Neph,S., Johnson,A.K., Sullivan,S.T., Sabo,P.J., Neri,F.V., *et al.* (2019) Mapping and dynamics of regulatory DNA in maturing Arabidopsis thaliana siliques. *Front. Plant Sci.*, **10**.

3. Pajoro,A., Madrigal,P., Muiño,J.M., Matus,J.T., Jin,J., Mecchia,M.A., Debernardi,J.M., Palatnik,J.F., Balazadeh,S., Arif,M., *et al.* (2014) Dynamics of chromatin accessibility and gene regulation by MADS-domain transcription factors in flower development. *Genome Biol.*, **15**, R41.

4. Cumbie,J.S., Filichkin,S.A. and Megraw,M. (2015) Improved DNase-seq protocol facilitates high resolution mapping of DNase I hypersensitive sites in roots in Arabidopsis thaliana. *Plant Methods*, **11**, 42.

5. Wu,L., Shang,G., Wang,F., Gao,J., Wan,M., Xu,Z. and Wang,J. (2022) Dynamic chromatin state profiling reveals regulatory roles of auxin and cytokinin in shoot regeneration. *Dev. Cell*, **57**, 526-542.e7.
